# Ca^+^ activity maps of astrocytes tagged by axoastrocytic AAV transfer

**DOI:** 10.1126/sciadv.abe5371

**Published:** 2022-02-09

**Authors:** Leonidas Georgiou, Anaí Echeverría, Achilleas Georgiou, Bernd Kuhn

**Affiliations:** Okinawa Institute of Science and Technology Graduate University, Okinawa, Japan.

## Abstract

Astrocytes exhibit localized Ca^2+^ microdomain (MD) activity thought to be actively involved in information processing in the brain. However, functional organization of Ca^2+^ MDs in space and time in relationship to behavior and neuronal activity is poorly understood. Here, we first show that adeno-associated virus (AAV) particles transfer anterogradely from axons to astrocytes. Then, we use this axoastrocytic AAV transfer to express genetically encoded Ca^2+^ indicators at high-contrast circuit specifically. In combination with two-photon microscopy and unbiased, event-based analysis, we investigated cortical astrocytes embedded in the vibrissal thalamocortical circuit. We found a wide range of Ca^2+^ MD signals, some of which were ultrafast (≤300 ms). Frequency and size of signals were extensively increased by locomotion but only subtly with sensory stimulation. The overlay of these signals resulted in behavior-dependent maps with characteristic Ca^2+^ activity hotspots, maybe representing memory engrams. These functional subdomains are stable over days, suggesting subcellular specialization.

## Teaser

Virus transfer from axons to astrocytes helps spy on their communication and reveal behavior-dependent astrocytic Ca^2+^ hotspots.

## INTRODUCTION

The past 30 years of research expanded our understanding of astrocytes from “support cells” to active participants in neuronal information processing. Astrocytes are glial cells that can translate local neuronal inputs into functional outputs via Ca^2+^ signaling cascades. Intracellular calcium concentration ([Ca^2+^]_i_) elevation in astrocytes can be induced by diverse inputs, including neurotransmitters released by minimal axon stimulation (*1*–*3*) and volume-transmitted neuromodulators (*4*–*7*). [Ca^2+^]_i_ elevation can modulate a plethora of functions, including synaptic activity (*8*–*10*), and energy supply (*9*, *11*).

However, how astrocytes communicate with neurons under physiological conditions is poorly understood. The active role of astrocytes in information processing was largely established by ex vivo studies focusing on somatic Ca^2+^ signals. A series of controversies argued that these slow (tens of seconds), widespread signals are incompatible with fast and localized modulation of synapses and blood vessels. They questioned how a cell-wide, on-off mode of signaling can mediate such diverse inputs and outputs (*9*, *12*, *13*). In addition, they pointed to confounding factors altering Ca^2+^ signals such as pathological experimental conditions (*14*), the young age of animals used (*15*), and anesthesia (*16*).

Technical advances offered solutions to these controversies and unearthed previously unidentified challenges. Genetically encoded calcium indicators (GECIs) targeting astrocyte plasma membranes revealed fast and localized Ca^2+^ microdomain (MD) activity in fine astrocytic processes. The spatiotemporal characteristics of these Ca^2+^ MD signals are compatible with neuronal and vascular dynamics (*9*, *17*–*21*). Cells use the diverse spatiotemporal dynamics of [Ca^2+^]_i_ and the associated signaling network for versatile control of cellular processes (*22*). The diversity of astrocytic Ca^2+^ signals and their dynamics could explain how astrocytes can elicit such varied responses in the brain. Now, automated, event-based analysis tools such as Automatic Quantification and Analysis (AQuA) (*23*) enable unbiased characterization of these spatially unfixed, size-varying Ca^2+^ MDs, overcoming the limitations of traditional, region of interest (ROI)–based methods. Furthermore, astrocyte signaling can be investigated under physiological conditions, at high spatiotemporal resolution using in vivo two-photon (2P) microscopy and sensory-driven neuronal stimulation in awake animals (*20*). However, whether physiological, sensory-driven neuronal inputs are sufficient to drive Ca^2+^ MD signals in astrocyte compartments remains controversial.

While some groups report reliable astrocyte Ca^2+^ responses to sensory-driven neuronal stimulation (*20*, *24*–*26*), others contradict it (*4*, *6*, *27*–*29*). Sensory-driven neuronal inputs should be sufficient to drive [Ca^2+^]_i_ elevations in astrocytes if they respond to minimal axon stimulation as reported by some studies (*1*–*3*) and contradicted by others (*28*, *30*). Sensory-driven responses might be subtle and dependent on the behavioral state of the animal. For example, locomotion in combination with visual stimuli synergistically enhanced Ca^2+^ responses in primary visual cortex astrocytes (*4*, *5*). Long-range cholinergic and noradrenergic neuromodulator projections might be necessary to enhance or enable local, sensory-induced Ca^2+^ MD signals in astrocytes. To complicate things further, astrocytes are heterogeneous cells that can discriminate between neuron subtypes and differentially modulate their activity (*31*). Different regions of an astrocyte may act as independent loci that differentially sense and modulate local neural activity (*1*, *7*, *27*). Such subdomains may act as independent units in processing information.

Although it is becoming evident that astrocytes are circuit-specific elements (*32*), currently, there are no good tools available for studying them in the context of the neuronal circuit they are embedded in. Viral tracing has been traditionally used to study neuronal circuits. Recently, adeno-associated viruses (AAVs) have been shown to undergo anterograde, kinesin-mediated transport (*33*, *34*) and to transduce postsynaptic neurons (*35*). This transfer depends on synaptic activity (*36*). So far, AAVs are mainly used as powerful transfection vectors widely used in gene therapy eliciting stable, long-term gene expression and minimal pathogenic side effects (*37*). Anterograde axoastrocytic transfer could have a major impact in deciphering circuit-specific neuron-astrocyte interactions and open previously unexpected frontiers in AAV-based gene therapy in the brain.

Here, we developed a next-generation platform to investigate astrocyte Ca^2+^ MD activity in behaving mice. First, we show that AAV2 serotype 1 (AAV1) injected in the thalamus can transfer anterogradely to cortical, second-order astrocytes and neurons. We used axoastrocytic AAV transfer to study layer 2/3 (L2/3), barrel cortex (BX) astrocytes embedded in the vibrissa thalamocortical circuit. Second, we characterized the amplitude, duration, size, and frequency of Ca^2+^ MD signals in the context of vibrissa stimulation and locomotion under physiological conditions at the single-astrocyte level. To do that, we combined sparse labeling of astrocytes with membrane-tagged GECIs, automated, event-based analysis tools, and high-resolution 2P microscopy. Third, we investigated how Ca^2+^ MD signals are spatially mapped in single astrocytes and whether they exhibit subdomains that are stable over time.

## RESULTS

### Anterograde axoastrocytic AAV transfer

Given the proximity of astrocyte processes to synaptic terminals (*38*) and that AAVs readily transduce astrocytes, we hypothesized that AAVs can undergo anterograde transport also to astrocytes. To test this hypothesis, we used AAV1, which was previously shown to exhibit robust anterograde trans-synaptic spread properties (*35*, *36*) in the vibrissa thalamocortical system of mice. The ventral posterior medial nucleus (VPM) of the vibrissa somatosensory system innervates primarily L4 and L6a and sends less dense projections to other layers including L2/3 of BX (*39*).

**Fig. 1.**
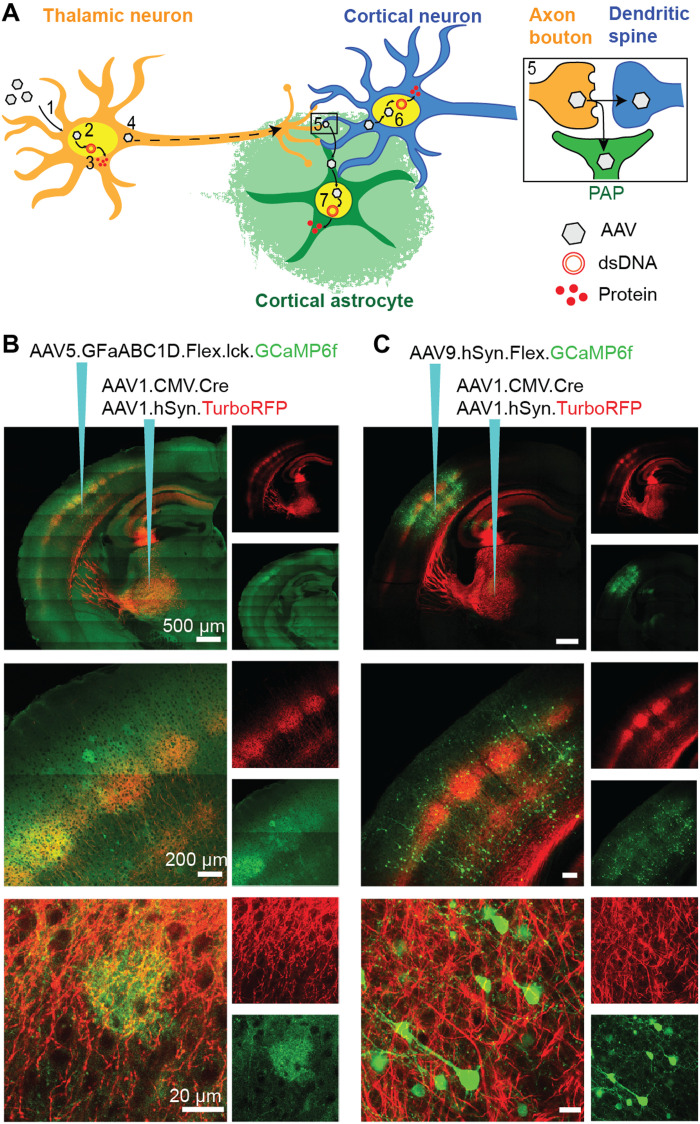
AAV1 transfer from VPM thalamocortical neurons to BX astrocytes and neurons. (**A**) Anterograde AAV transfer hypothesis: (1) AAVs enter neurons at the injection site by endocytosis. (2) Some AAV particles enter the nucleus and (3) release their single-stranded DNA (ssDNA), which is converted to double-stranded DNA (dsDNA) concatemers. The transgene is transcribed and then translated into proteins. (4) A small number of AAV particles are transported anterogradely along the axon to the terminals where (5) they are released and enter adjacent postsynaptic dendrites and perisynaptic astrocytic processes (PAP). Then, they enter the nuclei of (6) neurons and (7) astrocytes and release their ssDNA, which eventually leads to protein synthesis. (**B**) Intersectional AAV injection strategy for Cre-dependent GCaMP6f (green) labeling of BX astrocytes and TurboRFP (red) labeling of VPM neurons (red), 3 weeks after injection. (**C**) Intersectional AAV injection strategy for Cre-dependent GCaMP6f (green) labeling of BX neurons and TurboRFP (red) labeling of VPM neurons (red), 2 weeks after injection.

First, we tested whether AAV1 injections in VPM can lead to transduction of BX astrocytes and neurons (Fig. 1A). We coinjected AAV1 delivering the Cre gene under the cytomegalovirus promoter (CMV) AAV1-CMV-Cre and AAV1delivering the gene of the red chromophore TurboRFP under the human synapsin promoter (hSyn) AAV1-hSyn-TurboRFP in VPM. If a small number of AAVs are transported anterogradely, then we would expect ubiquitous, CMV promoter–driven expression of Cre-recombinase in BX astrocytes and neurons that can in turn engage the flip-excision (FLEx) switch to drive conditional gene expression (*40*). AAV1-hSyn-TurboRFP was used to mark the thalamocortical axon projections.

To test whether Cre was present in BX astrocytes, we additionally injected AAV5-GFaABC1D-FLEx-lck-GCaMP6f in BX to induce Cre-dependent expression of the GECI GCaMP6f selectively in astrocyte plasma membranes (Fig. 1B). This intersectional approach led to sparse labeling (~4 × 10^−6^ cells/μm^3^) of L2/3 BX astrocytes 3 weeks after AAV injections (Fig. 1B and fig. S1, A to C). We observed the highest density of astrocytes in L4 (19 × 10^−6^ cells/μm^3^) (Fig. 1B and fig. S1, A to C), the principal projection layer of the VPM as indicated by the density of TurboRFP-labeled thalamocortical axons (fig. S1, A, B, D, and E). The GFaABC1D promoter induced astrocyte-specific gene expression, the FLEx system allowed conditional expression in a few astrocytes expressing Cre recombinase, and the lck tag led to membrane tagging of GCaMP6f, revealing the cloud-like morphology of astrocytic nanoscopic processes (Fig. 1B and figs. S1A and S2A) (*18*). TurboRFP labeled thalamocortical axon projections to reveal their characteristic barrel-like projection patterns (*39*) expected in L4 of BX (Fig. 1, B and C, middle). All AAV injections (in BX and VPM) were performed during one surgery. We then tested whether Cre was also present in BX neurons. To do so, we instead injected AAV9-hSyn-FLEx-GCaMP6f in BX. We used the neuron-specific hSyn and the FLEx system to drive neuron-specific and Cre-dependent GCaMP6f expression. This intersectional strategy led to BX neuron labeling (Fig. 1C and fig. S1, D and E). We found the highest density of neurons in L4 (~11 × 10^−6^ cells/μm^3^) and L5/6 (~9 × 10^−6^ cells/μm^3^) (fig. S1F). In both preparations, no obvious TurboRFP^+^ cell bodies were detected in BX. So, the intersectional approaches resulted in sparse but bright labeling of L2/3 astrocytes and neurons (~3 × 10^−6^ cells/μm^3^) of the BX (fig. S1).

To confirm that the labeled cells were astrocytes or neurons, we used antibody labeling against S100β (Ca^2+^-binding protein concentrated in astrocytes; fig. S2A) and NeuN (neuronal nuclear antigen; fig. S2C), respectively. 94% of GCaMP6f^+^ BX cells were S100β^+^ in brains injected with AAV5-GFaABC1D-FLEx-lck-GCaMP6f (fig. S2B). Similarly, 97% of GCaMP6f^+^ BX cells were NeuN^+^ in brains injected with AAV9-hSyn-FLEx-GCaMP6f (fig. S2D).

**Fig. 2.**
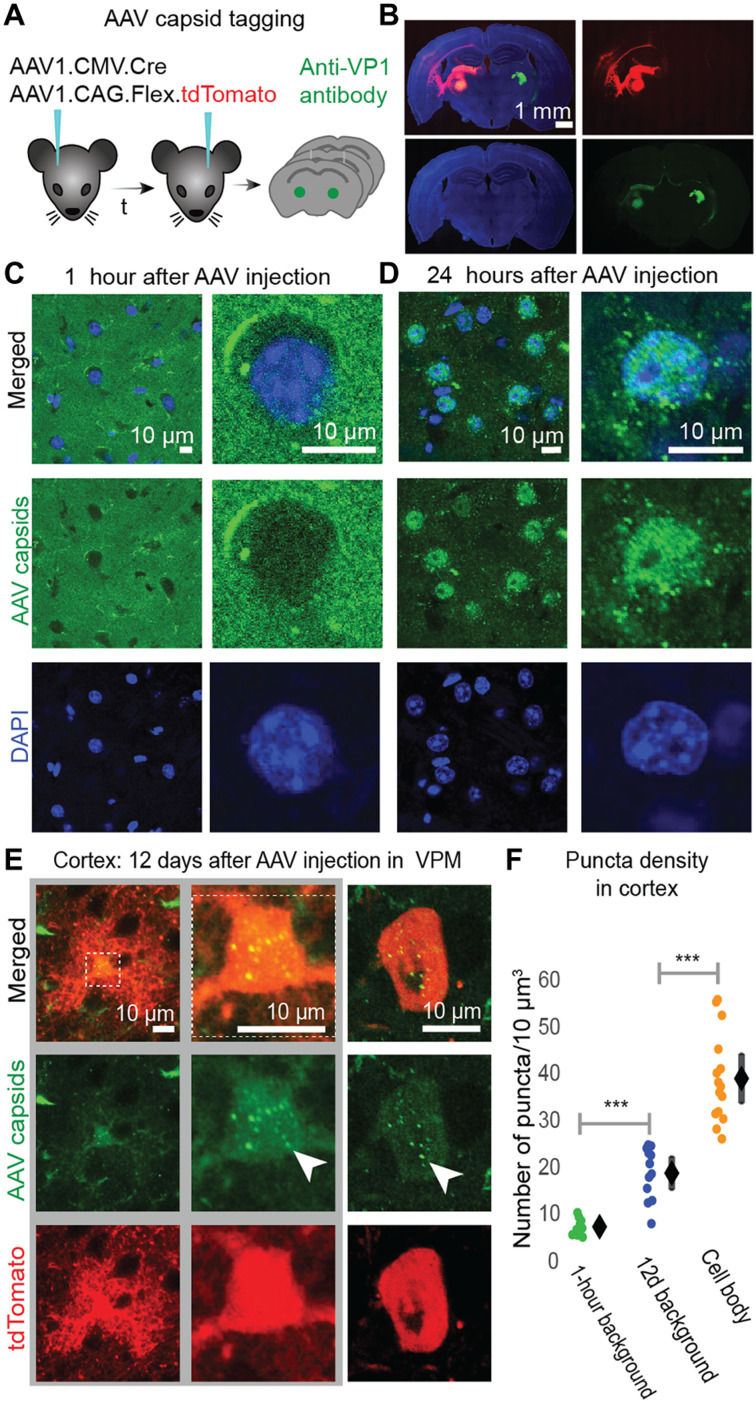
Antibody-labeled AAV1 capsids localize in VPM and BX cell bodies following AAV1 injection in VPM. (**A**) Schematic of AAV1 antibody-tagging experiment. One AAV1 injection [AAV1-CMV-Cre + AAV1-CAG-FLEx-tdTomato was performed in VPM (left) and one in the contralateral VPM (right, control) of the same mouse; the time delay (*t*) between each injection was either 24 hours (*n* = 3 mice) or 12 days (*n* = 3 mice)]. Within 1 hour of the second injection, animals were perfused and AAV capsids were tagged using anti-VP1 antibodies. (**B**) Coronal slice showing the two injection (in VPM) sites used in the experiment: 12 days after injection (left) and within 1 hour after injection (right). tdTomato (red) expression can be seen in VPM 12 days after AAV injection (left) but not in VPM within 1 hour of AAV injection (right), anti-VP1 antibody labeling of AAV capsids (green), and 4′,6-diamidino-2-phenylindole (DAPI) labeling of cell nuclei (blue). (**C**) AAV capsids (green) do not colocalize with VPM cell nuclei (blue) within 1 hour of AAV injection there. (**D**) AAV capsids (green) colocalize with VPM cell nuclei (blue) within 24 hours of AAV injection there. (**E**) AAV capsids (green puncta) are found in BX astrocyte (left, middle) and neuron (right) cell bodies expressing tdTomato (red) 12 days after AAV1 injection in VPM. White arrows indicate puncta. (**F**) Green, fluorescent puncta density in BX (L2/3 and L4) cell bodies (right) and BX background (area outside tdTomato^+^ cell bodies) 1 hour (left) or 12 days (middle) after AAV injection in ipsilateral VPM (1 hour background: 8.1 ± 1.2, *n* = 10 cells; 12d background: 19.6 ± 3.5, *n* = 12 cells; 12d cell bodies: 39.8 ± 5.3, *n* = 15 cells). Mean ± 95% CI. Unpaired two-tailed *t* test with significance threshold set to *P* < 0.05. ***P < 10^−6^.

Next, we investigated whether AAVs injected only in VPM can transduce astrocytes and neurons in BX. Because we suspected that only a small number of AAVs transfer from VPM to BX cells, we used vectors carrying genes encoding fluorescent proteins under control of the strong, ubiquitous CMV early enhancer/chicken β-actin (CAG) promoter (*41*). We injected (i) AAV1-CAG-GCaMP6f and AAV1-hSyn-TurboRFP (fig. S1A), (ii) AAV1-CMV-Cre and AAV1-CAG-FLEx-eGFP (enhanced green fluorescent protein) (fig. S1B), (iii) AAV1-CMV-Cre and AAV1-CAG-FLEx.tdTomato (Fig. 2, A to E), or (iv) AAV1-CMV-Cre, AAV1-CAG-FLEx-GCaMP6f, and AAV1-hSyn-TurboRFP (fig. S6, A and B) in VPM. These injections led to both neuron and astrocyte labeling (assessed by morphology) in BX. Therefore, a second cortical injection (intersectional strategy), the Cre-FLEx system, or a specific fluorescent protein being expressed is not required. However, a single-injection strategy relies on the use of very strong promoters, like the CAG promoter, and cannot be used in its current form for cell type–specific labeling. Therefore, we focused on intersectional approaches for the functional probing of astrocytes in this study because we could specifically label astrocytes while having a lower risk of toxicity induced by gene overexpression. Together, these experiments provide indirect evidence that a small number of AAVs transfer anterogradely from VPM thalamocortical axons to BX astrocytes and neurons.

To show direct evidence of anterograde AAV1 transfer to BX cells, we antibody-tagged AAV capsids (anti-VP1): First, we coinjected AAV1-CMV-Cre and AAV1-CAG-FLEx-tdTomato only in VPM because this strategy led to tdTomato-labeled astrocytes and neurons in BX. We perfused the animals 1 hour, 24 hours, or 12 days after the injection and then labeled brain slices with anti-VP1 (Fig. 2, A and B). One hour after AAV1 injection, AAV capsids dispersed around the injection site but did not enter the cell nuclei (Fig. 2C). Within 24 hours after injection, AAVs permeated the cell nuclei at the injection site (Fig. 2D). Twelve days after AAV injection in VPM, anti-VP1 labeled slices showed puncta in ipsilateral BX. We quantified the density of anti-VP1 tagged puncta in (i) BX cell bodies, (ii) the local background surrounding these cell bodies [12-day (12d) background], and (iii) the contralateral BX for nonspecific antibody labeling. Twelve days after injection in VPM, a small number of AAV capsids were found in tdTomato^+^ astrocytes and neurons in BX (Fig. 2E). There was a 103% (*P* < 10^−6^) higher density of anti-VP1–tagged puncta in cortical cell bodies compared to their local background (12d background, Fig. 2F). A similar pattern was observed in both astrocytes and neurons—identified on the basis of their morphology (Fig. 2E). This local background capsid density (12d background) was 142% higher (*P* < 10^−5^) than the density of puncta in contralateral (control) BX (Fig. 2F). The unexpectedly high density of puncta in the 12d background (compared to contralateral BX) is likely due to AAV capsids residing in cell bodies and neurites not expressing detectable levels of tdTomato (Fig. 2F).

In summary, we showed that AAV1 particles injected in VPM transfer with low probability anterogradely to BX astrocytes and neurons. Anterograde axoastrocytic transfer could be used to study astrocytes embedded in specific neuronal circuits.

### Astrocyte Ca^2+^ MD activity in behaving mice

**Fig. 3.**
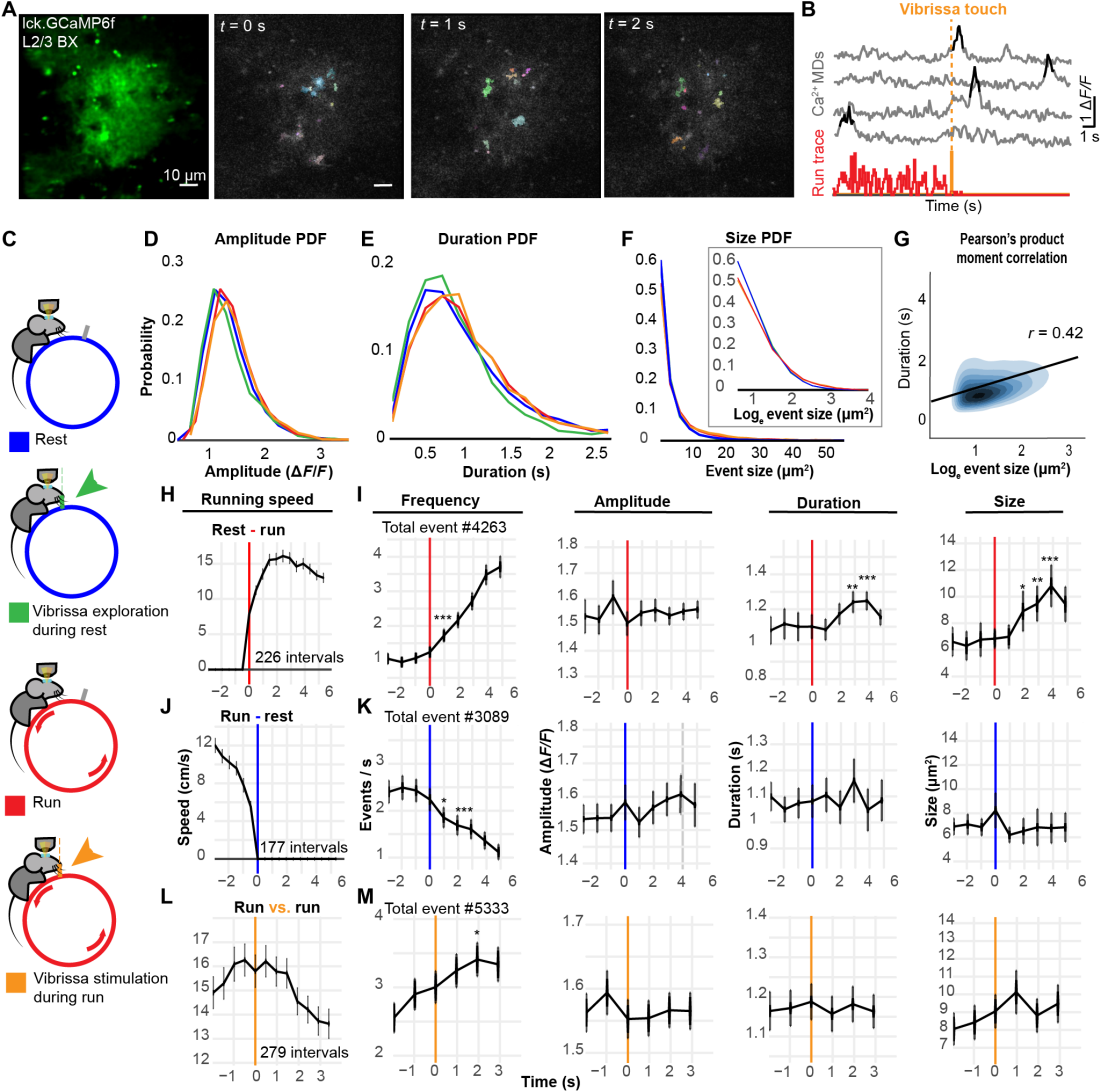
Astrocyte Ca^2+^ MD signal characteristics during behavioral states and vibrissa stimulation. (**A**) Single astrocyte in L2/3 of barrel cortex labeled with membrane-tagged GCaMP6f and imaged with 2P microscopy in awake mice (left), followed by examples of Ca^2+^ MD signals (colored footprints) automatically extracted with AQuA over 2 s. (**B**) Example Ca^2+^ MD signal traces (gray) and events (black) extracted by AQuA during run (red) and vibrissa touch (orange). (**C**) Schematic of four states explored (three mice, nine astrocyte recordings). Probability distributions of Ca^2+^ MD signal: (**D**) amplitudes (Δ*F*/*F*), (**E**) durations (s), and (**F**) sizes (μm^2^) and log_e_ sizes. Each line represents the distribution of event characteristics extracted during the four behavioral states of all (*n* = 9) recordings: rest (blue; *n* = 29,637), run (red, *n* = 15,123), vibrissa exploration (green, *n* = 3121), and within 1.5 s of vibrissa stimulation (orange, *n* = 2906). (**G**) Density plot and Pearson’s product moment correlation (*r*) between log_e_ event sizes, and their durations (*n* = 50,787 events). (**H**) Average running speed during rest to run state transitions (*n* = 226). (**I**) Mean frequency (events/s), amplitude, duration, and size of MD signals (*n* = 4263) during rest to run state transition. (**J**) Average running speed during run to rest state transitions (*n* = 177). (**K**) Mean frequency, amplitude, duration, and size of MD signals (*n* = 3089) during run to rest state transition. (**L**) Average running speed during continuous running, intercepted by vibrissa stimulus (*n* = 279). (**M**) Mean frequency, amplitude, duration, and size of MD signals (*n* = 5333) during vibrissa stimulation. Kruskal-Wallis test with Bonferroni correction (*P < 0.05, **P < 0.01, and ***P < 0.001, compared to activity at −1 s from state transition). Mean ± 95% CI. 1-s-bin average (black traces).

Next, we developed a platform that enables the detailed characterization of Ca^2+^ MD activity in the fine processes of single astrocytes under physiological conditions. To do so, we combined axoastrocytic AAV transfer with state-of-the-art genetic, imaging, and analysis tools. To label BX astrocytes embedded in the vibrissal thalamocortical circuit, we injected AAV1-CMV-Cre into VPM and AAV5-GFaABC1D-FLEx-lck-GCaMP6f into BX (Fig. 1B). This intersectional approach led to sparse labeling of L2/3 astrocytes (Fig. 3A). Sparse fluorescent labeling is ideal for high-resolution imaging of single astrocytes because it minimizes unwanted background fluorescence. To selectively monitor the fast, near-membrane Ca^2+^ MD activity at fine astrocytic processes, we tagged GCaMP6f to the astrocytic plasma membrane using the lck membrane-targeting motif (*17*–*19*). We then used 2P microscopy (*42*) to detect Ca^2+^ activity with high spatiotemporal resolution in behaving, adult mice. We took advantage of the high-photon collection efficiency of high–numerical aperture (NA) objectives in conjunction with elongating the point spread function (PSF) of excitation by underfilling the back aperture of the objective to enhance signal-to-noise ratio, minimize photobleaching, and sample a volume with an axial length of ~5 μm (*43*). We recorded at 30.9-Hz frame rate using a resonant scanner. This imaging strategy enables long-duration (>1 hour), volumetric imaging at high spatiotemporal resolution in contrast to the slow-acquisition rate of three-dimensional (3D) imaging (*1*). To analyze Ca^2+^ MD dynamics in an automated, unbiased, event-based way, we used AQuA (*23*). To induce sensory-driven neuronal stimulation during locomotion, we incorporated a pole into a cylindrical treadmill, positioned to intercept and touch the vibrissae contralateral to the injection site as the animal runs (movie S1).

We used the platform to investigate near-membrane Ca^2+^ MDs in single astrocytes (Fig. 3B) during rest (29,637 events), locomotion (15,123 events), vibrissa stimulation (2906 events), and vibrissa exploration (3121 events) (nine astrocyte recordings, three mice) (Fig. 3C and fig. S5A). Vibrissa stimulation is defined as the 1.5-s period following contralateral vibrissa touch during locomotion. Vibrissa exploration refers to vibrissa touch periods while the animal is at rest. These two behavioral states represent substates of run and rest states, respectively. We characterized Ca^2+^ MD signal amplitudes (Δ*F/F*), sizes (μm^2^), durations (s), and frequency (number of events per time) during four behavioral states of the animal (rest, run, vibrissa exploration, and vibrissa stimulation). We found that the amplitude (mean Δ*F/F* = 1.6) (Fig. 3D) and duration (mean = 1.1 s) (Fig. 3E) of Ca^2+^ MD signals can be approximated as skewed normal distributions (fig. S4, A and B). There was no obvious change in the distribution of event amplitudes (Fig. 2D and fig. S4A) with the state of the animal. The distribution of event durations was slightly shifted to the right during run compared to rest suggesting a small increase in event duration during locomotion (Fig. 3E and fig. S4B). We detected ultrafast Ca^2+^ signals with ≤300-ms duration (temporal resolution limit of the recording after filtering is 200 ms). With our specific imaging and analysis settings, 2.8% ± 1.3 confidence interval (CI), ± 1.6 SD of events were ultrafast (≤ 300 ms), with an average Δ*F/F* amplitude of 1.7 ± 0.18 CI, ± 0.22 SD, and a mean size of 3.04 μm^2^ ± 0.24 CI, ± 0.29 SD. The size (spatial spread) distribution of all events can be described as a heavy-tailed distribution (Fig. 3F), best fitted by a lognormal or power law distribution (fig. S4C). We found a positive correlation between event size and duration (*r* = 0.42; Fig. 3G). Overall, we observed subtle changes in the distribution of event durations and sizes between rest and run but not with vibrissa stimulation or exploration.

We then assessed how Ca^2+^ MD signal characteristics change with the onset of behavior state transitions (Fig. 3, H to M). We compared changes in event characteristics initiated before and after the onset of locomotion (Fig. 3, H and I), offset of locomotion (Fig. 3, J and K), and onset of vibrissa stimulus (Fig. 3, L and M). With the onset of locomotion, we found a 290% increase in the mean event frequency (rest: 0.95 to run: 3.7 events/s, *P* < 2 × 10^−50^). We also observed a 65% increase in mean event size (rest: 6.6 to run: 10.8 μm^2^, *P* < 2 × 10^−4^) and 13% increase in duration (rest: 1.10 s to run: 1.25 s, *P* < 0.02) (Fig. 3I). There was no significant change in mean amplitude of events (Δ*F/F* = 1.54 to 1.61 during 1-s intervals, *P* > 0.05 for all pairs). With the offset of locomotion (Fig. 3J), there was a 54% decrease in the frequency of events (run: 2.4 to rest: 1.1 events/s, *P* < 2 × 10^−16^) but no significant change in amplitude, duration, and size (Fig. 3K). Therefore, locomotion increases event frequency, size, and duration but not amplitude. A sustained frequency of ~2.4 events/s was maintained during running, but the duration and size of events returned to baseline, seconds after the onset of locomotion. There was a 17%, graded increase (*P* < 0.05) in the mean frequency of events following vibrissa stimulation during running (Fig. 3M). However, this may be attributed to fluctuation in the mean running speed (Fig. 3L), because the frequency of events started increasing before the onset of vibrissa stimulation (Fig. 3M). Alternatively, it could reflect an enhanced Ca^2+^ activity response induced by synergistic neuromodulator and neurotransmitter inputs during locomotion and sensory stimulation (*4*, *5*). There was no fast, transient change in any event characteristics during vibrissa stimulation (Fig. 3M). This suggests that vibrissa stimulation triggers subtle Ca^2+^ responses that drown in the ongoing activity of the active awake brain. Locomotion predominantly influences event frequency and size (fig. S5B). There was no significant increase in the mean duration of events during locomotion suggesting that the increase in duration is a response to the acceleration phase of locomotion. We found no significant change (*P* > 0.05) in mean Ca^2+^ MD signal characteristics during vibrissa exploration (fig. S5C), but we found a 15% increase (*P* < 0.05) in Ca^2+^ event frequency with vibrissa stimulation (fig. S5D).

To confirm that vibrissa stimulation induces neuronal activity in BX, we recorded the Ca^2+^ activity of thalamocortical axons during different behavioral states. We coinjected AAV1-CMV-Cre, AAV1-hSyn-TurboRFP, and AAV1-CAG-FLEx-GCaMP6f only in VPM (*n* = 3 mice; fig. S6A), which leads to colabeling of thalamocortical axons with GCaMP6f and TurboRFP, while BX astrocytes were only labeled with GCaMP6f (fig. S6B). This allowed us to simultaneously record the Ca^2+^ activity of single astrocytes and thalamocortical axons in BX (movie S2). Thalamocortical axons analyzed outside the territory of labeled astrocytes (*n* = 3 recordings; fig. S6B) responded to the onset of locomotion (174 intervals) with transient [Ca^2+^]_i_ elevations starting before the onset of locomotion (~0.5 s) and reaching peak event frequency (~129% increase in event/s from rest baseline) ~0.3 s after locomotion started (fig. S6C). Following this acceleration phase, Ca^2+^ activity decreased to a running baseline that was ~55% higher than rest baseline (fig. S6C). Running baseline Ca^2+^ activity decreased to rest baseline activity with the offset of locomotion (fig. S6D). Following the onset of vibrissa stimulation (~0.2 s), axons responded with a sharp increase (~97%) in Ca^2+^ activity from mean run baseline (756 intervals) (fig. S6E). This confirmed that the subtle vibrissa stimulus introduced by a single pole drives thalamocortical activity in the vibrissa somatosensory system as expected. We found no change in astrocytic Ca^2+^ activity (fig. S6F, 6091 events and 756 intervals) with vibrissa stimulation (fig. S6G). While vibrissa stimulation by a single pole was sufficient to induce axonal stimulation in the BX, it may have been too weak for inducing changes in astrocyte Ca^2+^ MD signaling.

We then tested whether higher-frequency vibrissa stimulation could elicit more reliable astrocyte Ca^2+^ activity. To do that, we covered the circumference of the treadmill with 23 poles (fig. S7A). The idea was that vibrissa would be stimulated by multiple poles as the animal was running, mimicking high-frequency vibrissa stimulation in a physiological setting. We hypothesized that a higher-frequency stimulation (23 poles) would lead to increased Ca^2+^ MD signal frequency, duration, size, and amplitude compared to single or no vibrissa stimulation during locomotion.

We characterized Ca^2+^ MD signals of the same astrocytes (*n* = 3 astrocytes, 3 mice, 12 recordings) during locomotion on the 23-pole treadmill (6 recordings) and 1-pole treadmill (6 recordings) (fig. S7A). Ca^2+^ event characteristics were normalized to the mean rest state activity of the respective recording. Compared to locomotion without vibrissa stimulation, increased frequency of vibrissa stimulation (23-pole) was associated with a small increase in event duration (17%, *P* < 0.04) and size (17%, *P* < 0.02) and a trend of increase of frequency [70%, not significant (n.s.), *P* = 0.06] but not amplitude. Notably, comparing stimulation by 1 pole with 23 poles per revolution, we found a trend of higher Ca^2+^ event duration (9%, n.s., *P* = 0.2) and frequency (40%, n.s., *P* = 0.3) and an increase in size (29%, *P* < 0.02) (fig. S7B).

This shows that physiological vibrissa stimulation subtly alters the Ca^2+^ MD activity in the fine processes of L2/3, BX astrocytes. Increasing the frequency of vibrissa stimulation is associated with subtle increase in Ca^2+^ MD signal duration, size, and frequency but not amplitude.

### Astrocyte Ca^2+^ activity maps

Astrocytes have a rich repertoire of Ca^2+^ sources and sinks that interact with a heterogeneous environment to control Ca^2+^ fluxes in highly regulated ways. The spatiotemporal characteristics of Ca^2+^ fluxes, coupled to a diverse signaling network, can drive variable responses (*19*, *22*, *44*). Different astrocytic processes might differentially interact with local synapses enabling heterogeneous and local processing. This idea is supported by recent reports of heterogeneous Ca^2+^ activity patterns in single astrocytes (*1*, *7*, *27*). This heterogeneous environment of each astrocyte is stable over time: The neuronal network and its connections barely change over months in cortex (*45*). We therefore expected that also the downstream intracellular arrangement is lasting and hypothesized that BX astrocytes exhibit stable-over-time Ca^2+^ hotspot maps.

**Fig. 4.**
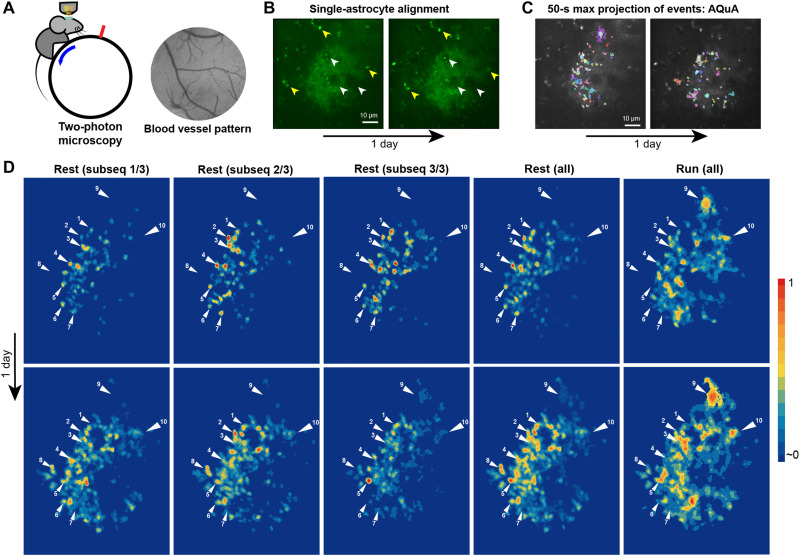
Astrocyte Ca^2+^ hotspot stability over time. (**A**) Single astrocytes were recorded using 2P microscopy in head-fixed mice free to move on a treadmill (left). The coordinates of single astrocytes were mapped in relation to the blood vessel pattern obtained using wide-field microscopy (right). (**B**) Z-plane alignment of the same astrocyte between days by aligning morphological features: astrocyte morphology, blood vessels passing through the astrocyte (white pointers) and fluorescent puncta patterns (yellow pointers). (**C**) 50-s maximum projection of Ca^2+^ signals extracted with AQuA of the same astrocyte recorded over 1 day. (**D**) Normalized hotspot maps of an astrocyte recorded 1 day apart during rest and running (run all). Activity heatmaps during rest state (rest all) were split into three equitemporal subsequences (rest subsequence, 20 min each). Similar hotspot patters can be observed between subsequences within day (within row), between days (top row versus bottom row), and during rest and run (white pointers 1 to 10 indicate example hotspot loci). Some hotspots are appearing or disappearing over time or during different behavioral state (rest versus run). All heatmaps were normalized to their maximum pixel value.

To test this hypothesis, we performed long-duration recordings (22 to 106 min) in single astrocytes (*n* = 6) labeled with membrane-tagged GCaMP6f in behaving mice (Fig. 4A). To investigate hotspot stability over days, we recorded three of these astrocytes over consecutive days (total of 9 recordings). To localize the same astrocytes over consecutive days, we used the blood vessel pattern on the brain surface (Fig. 4A), vessels perforating the astrocyte, and autofluorescent puncta patterns (Fig. 4B). Ca^2+^ MD signals were extracted with AQuA (Fig. 4C). The area of their footprints was summed in time to construct activity maps (Fig. 4D and figs. S8A and S10). Activity maps revealed regions of localized Ca^2+^ activity hotspots (Fig. 4D and figs. S8A and S10). This suggests that astrocytes exhibit subcellular heterogeneity in their Ca^2+^ activity patterns. If Ca^2+^ MD activity was randomly distributed, then a more homogeneous activity heatmap is expected. This can be illustrated by simulations constructed on the basis of real Ca^2+^ signals randomly distributed in masks corresponding to a respective astrocyte recording (fig. S8A). Natural heatmaps but not simulated random distributions of Ca^2+^ MD signals are characterized by hotspot patterns (fig. S8A), and their heatmaps exhibit different distributions in the frequency of pixel intensity values (fig. S8B).

To determine how long it takes to determine a reliable hotspot map, we split the video recordings into shorter subsequences and compared them to the overall activity heatmap of the recording (*n* = 5). This revealed that a 20-min recording shows about 85% similarity to the full-recording heatmap (fig. S9A). Therefore, ~20 min of recording can yield a good approximation of the astrocyte Ca^2+^ activity map. The progressive generation of a 20-min activity heatmap associated with rest and run states can be viewed in movie S3.

We then asked whether the hotspot patterns observed were stable over time. By comparing heatmaps generated from movie sequences of similar length (>17 min) while animals were at rest, we observed similar hotspot patterns over time (Fig. 4D, example arrows). We found a Pearson correlation coefficient (PCC) of 0.61 between subsequence heatmaps generated from the same astrocyte recording (*n* = 5 astrocyte recordings, >70 min each, three sequences each; fig. S9B, “Self”). The similarity between the real activity heatmap subsequences was significantly (*P* < 0.0001) higher than the similarity to their respective random distribution (PCC = 0.11, “Random”). There was significant (*P* < 0.0001) similarity (PCC = 0.50, “States”) between the heatmaps generated during rest and run states of the animal compared to random distributions (fig. S9B), with notable hotspot pattern changes associated with the state of the animal (Fig. 4D and fig. S10).

Are Ca^2+^ activity maps stable over days? Heatmaps generated from the same astrocyte recorded over subsequent days revealed similarities in their hotspot patterns (Fig. 4D). We found a PCC = 0.28 (“Days”) between heatmaps generated from subsequent day recordings that was significantly higher (*P* = 0.0001) compared to random distribution (fig. S9B).

## DISCUSSION

In summary, (i) AAV1 transfer anterogradely to astrocytes and neurons. (ii) Our experimental platform can be used to study single-astrocyte Ca^2+^ activity, in an unbiased way, at high spatiotemporal resolution, within a specific neuronal circuit (thalamocortical), for long durations (>1 hour), under physiological conditions, in behaving mice. (iii) With this platform, we characterized the spatiotemporal dynamics of Ca^2+^ MD signals in single astrocytes. We found that frequency, duration, and size of Ca^2+^ MD signals increased markedly with volitional locomotion. There was no change in these characteristics with sparse vibrissa stimulation, except for a slight increase in Ca^2+^ event frequency. Higher-frequency vibrissa stimulation resulted in subtle but significant increase in Ca^2+^ MD signal size and duration. Event amplitudes did not change in the behavioral contexts tested. In addition to previously reported Ca^2+^ MD activity, we find ultrafast Ca^2+^ MD activity (≤300 ms) that occurs on a spatial scale of neuronal spines and on a temporal scale of neuronal Ca^2+^ activity. (iv) Astrocytes exhibit behavior-dependent Ca^2+^ activity hotspot maps that are stable over days.

### AAV1 transfer anterogradely to astrocytes and neurons

Prior findings demonstrated anterograde AAV transfer to neurons (*35*, *36*). Here, we show that AAVs also transfer to astrocytes (Figs. 1 and 2 and fig. S1 to S3). Astrocytes were defined by morphological, molecular, and functional markers: Astrocytes exhibit a characteristic cloud-like morphology. These cloud-like cells expressed the astrocyte marker S100β (fig. S2). Fluorescence expression in these cells was driven by the astrocyte-specific promoter GFaABC1D. Last, these cells exhibited the characteristic Ca^2+^ MD activity seen in astrocytes (movie S1). In addition, we find AAV capsids in second-order labeled neurons and astrocytes. We used three lines of evidence to support axoastrocytic AAV transfer.

First, intersectional approaches demonstrated Cre-dependent expression of GCaMP in BX astrocytes and neurons (Figs. 1 and 2 and fig. S2 and S3). Given that AAV1-Cre was injected in the VPM, and Cre is required to trigger FLEx-dependent fluorescence expression, Cre-recombinase must have been transferred to the BX where AAV-GFaABC1D-Flex-GCaMP was injected. The conditional expression of GCaMP was in astrocytes expressing Cre-recombinase because fluorescence gene expression was driven by the astrocyte promoter GFaABC1D. Similarly, we observed neuron-specific labeling when fluorescence expression was driven by the hSyn promoter. Previously, Castle *et al.* (*34*) showed that AAVs can travel along axons driven by kinesin-2. Zingg *et al.* (*36*) showed that trans-synaptic AAV transport is mediated by synaptic transmission in glutamatergic and GABAergic, but not neuromodulatory, projections. Our evidence suggests that AAV1 particles transported via thalamocortical glutamatergic projections transfer to both astrocytes and neurons in the cortex.

A single astrocyte cradles and maintains ~140,000 synapses within its territory (*46*). AAVs escaping postsynaptic boutons could enter the fine perisynaptic astrocytic processes in addition to postsynaptic terminals. Most of the astrocytes and neurons labeled were in L4 (fig. S3), the major postsynaptic projection layer of the VPM. The distribution of labeled astrocytes in the cortical column suggests a positive relationship between the density of presynaptic inputs to astrocytes and the probability of Cre expression in these cells. We observed a higher density of astrocytes to neurons (1.7: 1) in L4 using intersectional injection strategies. It is important to note that density estimations are highly variable and heavily dependent on experimental conditions (accuracy of injection and real viral titer). Even so, there are six times more neurons than astrocytes in the cortex (*47*), suggesting that anterograde AAV1 transfer may more efficiently transduce astrocytes than neurons in the same circuit. Only a small amount of Cre-recombinase is needed to engage the FLEx switch (*48*) allowing intersectional strategies to be effective even if AAV transfer is a low-probability event. We always used AAV1 in the VPM because it was shown to undergo more efficient anterograde transfer compared to other tested serotypes (*35*).

Second, we found that BX astrocytes and neurons can be labeled by injecting AAVs carrying genes encoding fluorescent proteins only in VPM (fig. S1). BX cell labeling was dependent on the use of the very strong CAG promoter but not on the specific fluorescent protein expressed or the use of Cre recombinase. One explanation is that only a small number of AAVs eventually reach the postsynaptic cell nucleus, and high gene expression is needed to produce detectable levels of fluorescent proteins in the cell. While these single-injection strategies can be used to label postsynaptic astrocytes and neurons, it suffers from two important limitations: (i) It cannot be used for cell type–specific labeling in its current form and (ii) very strong promoters can lead to overexpression of genes, cell death, and pathology. The direct labeling of BX astrocytes and neurons with AAV1 injections only in VPM further suggests anterograde AAV1 transfer to astrocytes.

Third, we showed that AAV particles are transferred (Fig. 2, D to H). Previous studies showed indirect evidence of AAV transfer between neurons (*35*, *36*). Here, we expand these findings by showing direct evidence that AAV capsids can be found in second-order neurons and astrocytes in BX within 12 days of injection in VPM.

All evidence points to the idea that AAVs can transfer anterogradely across the tripartite synapse to astrocytes and neurons. However, we cannot rule out AAV spillover from axons captured by nearby astrocytic processes. Therefore, we prefer to call the transfer axoastrocytic over trans-synaptic.

Axoastrocytic AAV transfer is a new tool to study circuit-specific neuron-astrocyte interactions. It also reinforces the idea that AAVs are not as localized to the injection site as commonly believed but transfer to second-order cells. The spread of AAVs beyond the injection site and cell type of interest should be taken into consideration in scientific research or clinical gene therapy using these vectors.

In this study, we demonstrated anterograde axoastrocytic AAV transfer only in the VPM-BX circuit. To generalize these findings, more circuits must be investigated. Furthermore, the mechanisms of AAV transfer to astrocytes remains to be elucidated.

### Novel platform for studying single-astrocyte Ca^2+^ MD signals under physiological conditions

We then created a novel platform for studying single astrocytes embedded in the vibrissa thalamocortical circuit under physiological conditions. To do that, we combined axoastrocytic AAV transfer in combination with GECIs, advanced 2P microscopy, and AQuA. Axoastrocytic AAV transfer and sparse Cre-recombinase expression in postsynaptic astrocytes enabled circuit-specific investigations. Conditional expression of membrane-tagged GCaMP6f in astrocytes enabled Ca^2+^ sensing near plasma membranes. Fast, high-resolution 2P microscopy enabled subsecond, micrometer imaging of Ca^2+^ MD activity. Hour-long Ca^2+^ MD signal recordings were analyzed in an unbiased, event-based way using AQuA. We designed the platform to address the nuanced experimental conditions necessary to capture the physiology of fast neuron-astrocyte interactions. We believe that many of the controversies in the field are due to experimental conditions and limitations, such as (i) physiological conditions in brain slice experiments and choice of sensory, electrical, or optogenetic stimulation in in vivo experiments; (ii) difference in age of animals used; (iii) the use of anesthetics and the effect of different anesthetics; (iv) spatiotemporal resolution or signal-to-noise ratio used to investigate Ca^2+^ MD activity; (v) duration of imaging sessions to achieve significant results; (vi) differences between ROI and event-based analysis; and (vii) studying astrocyte-neuron interactions in a circuit-independent manner while astrocytes are thought to be circuit-specific elements (*32*).

Here, we used (i) in vivo experiments, where mice are exposed to physiological vibrissa stimuli while free to move at their own volition to minimize startle and other stress inducing confounding stimuli; (ii) adult mice; (iii) mice that are awake; (iv) recording of fast (subsecond) Ca^2+^ activity using resonant scanning volumetric imaging of individual, sparsely labeled astrocytes, recorded at single-micrometer resolution using membrane-bound GCaMP6f in fine astrocytic processes; GCaMP6f has faster kinetics than older versions of this GECI; (v) long (>1 hour) astrocyte recordings; (vi) automated, ROI-independent analysis of Ca^2+^ MD signal characteristics; and (vii) circuit-specific neuron-astrocyte interactions using axoastrocytic AAV transfer.

We used our platform to characterize Ca^2+^ MD signal characteristics (frequency, duration, size, and amplitude) during rest, locomotion, and vibrissa stimulation. We found that the mean duration of Ca^2+^ events detected was less than a fifth of previously reported mean durations in vivo and the fastest (200 ms) less than a third of the shortest events detected ex vivo (650 to 750 ms) (*2*). Such fast astrocytic Ca^2+^ MD activity strongly suggests that astrocytes can be actively involved in fast information processing in the brain. It will become crucial to take the ultrafast (≤300 ms) Ca^2+^ MD activity into account to evaluate neuron-astrocyte interaction. By extrapolating the trend of the lognormal and power law fit (fig. S4C) to the nanoscale (here experimentally unresolved), we predict frequent, ultrafast Ca^2+^ nanodomain (<1 μm^2^) signals.

### Astrocyte Ca^2+^ MD activity increases extensively with locomotion and subtly with vibrissa stimulation

Our findings indicate that astrocyte Ca^2+^ responses to neuronal circuit activity triggered by sensory stimulation are subtle (Fig. 3 and figs. S4, S5, and S7) and not as reliable as suggested by previous ex vivo experiments (*1*–*3*). They are also less reliable than Ca^2+^ activity detected in thalamocortical axons in BX (fig. S6). Given the rich baseline neuronal activity of the awake brain, it is likely that sensory stimulation only marginally perturbs the activity around the astrocyte. Increasing the frequency of sensory stimulation by introducing more poles to our setup increased frequency (trend), duration, and size but not amplitude of Ca^2+^ MD activity (fig. S7). These observations suggest that astrocyte Ca^2+^ MD activity is weakly coupled to physiological, vibrissa-induced neuronal stimulation and that this coupling increases with the frequency of vibrissa stimulation.

It is thought that the behavioral state of the animal, such as locomotion, can increase the gain of astrocytes to sensory stimuli (*4*, *5*). We detected only subtle increase in Ca^2+^ event frequency to vibrissa stimulation during volitional locomotion (Fig. 3M and fig. S5D). Furthermore, we did not detect reliable astrocyte Ca^2+^ MD signaling in response to nearby axon bouton activity as previously suggested by ex vivo experiments (movie S2) (*1*–*3*). Sensory inputs to astrocytes might be drowning in the activity driven by disproportionately more numerous inputs generated in an awake brain, in particular during locomotion.

We also showed that astrocytes reliably respond to volitional locomotion (Fig. 3I and figs. S4 and S5B), with increase in Ca^2+^ MD signal frequency, size, and duration, expanding on previous findings (*4*–*7*) and reinforcing the idea that astrocytes respond more readily to neuromodulators than neurotransmitters. The changes observed are similar to findings showing a biphasic response to startle induced locomotion, characterized by a fast inositol 1,4,5-trisphosphate receptor type 2 (IP3R2)-dependent and a delayed IP3R2-independent component (*17*). This suggests that the increase in duration and size of Ca^2+^ MD signals observed with the acceleration phase of locomotion might be mediated by an IP3R2-dependent mechanism.

### Astrocytes exhibit behavior-dependent, stable-over-time Ca^2+^ hotspot maps

The stability of astrocytic Ca^2+^ MD activity over time was previously shown by Stobart *et al.* (*49*) on a slow time scale (typical MD signal lasted 20 s) where the same ROIs within a wide field of astrocytes exhibited higher, stable Ca^2+^ MD activity to strong vibrissa stimulation (90 Hz) over months. Here, we show the existence of persistent Ca^2+^ MD activity maps in the fine processes of single astrocytes on a time scale down to 200 ms that are stable over days (Fig. 4, figs. S8 to S10, and movie S3). The similarity between activity heatmaps over time persisted despite challenges to perfect alignment of the z-plane. We partly overcame this limitation by elongating the PSF in the z-plane. The stability of Ca^2+^ activity hotspots is compatible with long-term information storage, as indicated by the stability of spines in vivo (*45*), and may be involved in memory traces or engrams of synaptic activity. Alternatively, they could represent hotspots of higher metabolic demand and stress. Certain hotspots appear to be modulated by the state of the animal. Different inputs to the cell might activate different MDs leading to hotspot pattern changes (*7*). The subcellular heterogeneity in astrocyte Ca^2+^ signaling is likely mediated by differences in the localization and activity of Ca^2+^ sources and sinks, such as plasma membrane channels, mitochondria, and endoplasmic reticulum. In addition, Ca^2+^ activity hotspots in astrocytes might correspond to regions of higher neuronal activity, leading to mitochondria recruitment in these regions (*7*), increased potassium uptake (*50*), or sensing of transmitters and modulators, which can lead to Ca^2+^ activity (*10*). The demonstration of local mRNA translation at astrocytic processes (*51*) and distinct gliotransmitter release pathways in single astrocytes (*52*) suggest diverse potential responses triggered by local Ca^2+^ activity.

#### Limitations

The limitations of this study are as follows: (i) Here, we showed that AAV1 particles transfer from VPM to cortical neurons and astrocytes. While our data suggest that the transfer occurs at the tripartite synapse, it could also be explained by local spillover. Furthermore, we do not show whether the transfer to astrocytes is synapse type specific as the transfer between neurons (*36*). (ii) It is important to note that the puncta detected might represent single or clusters of antibody-tagged capsids, making quantification of the number of capsids unreliable. We, therefore, reframe from drawing quantitative comparisons beyond the general claim of a larger presence of puncta in labeled BX cell bodies. (iii) The small total number of astrocytes and animals used for the in vivo studies limited the exploration of very small changes in Ca^2+^ activity. This limitation was compensated by extensive data extracted from continuous, hour-long recordings of each probed astrocyte. (iv) The small number of detected ultrafast events (2.8% ± 1.3 CI, ± 1.6 SD of all detected events) is most likely due to limitations of the signal-to-noise ratio of the recordings and the event detection threshold. In other words, the here detected frequency of ultrafast events is most likely not representing the physiological frequency of occurrences. With an increased signal-to-noise ratio, we expect a highly increased frequency of ultrafast events with diameters <2 μm. (v) The investigation was limited to only L2/3 BX astrocytes embedded in the VPM thalamocortical circuit. It is likely that astrocytes exhibit a heterogeneous profile of Ca^2+^ MD activity in different layers, brain regions, and specific neuronal circuits not investigated here. (vi) We did not investigate how Ca^2+^ signals propagate in space or how Ca^2+^ MD activity changes differ between different astrocytic subcompartments. (vii) We did not disentangle mechanisms of action using pharmacology or transgenic animals. These limitations should be overcome in future studies using the presented platform and using the results generated in this study as a physiological baseline. (viii) There was high variability between Ca^2+^ recordings of different astrocytes. Therefore, we refrain from making any conclusions about physiological heterogeneity between astrocytes, although we expect it.

#### Significance

The significance of the study is shown in the following: (i) The discovery of anterograde axoastrocytic AAV transfer can be used to study circuit-specific neuron-astrocyte interactions. The spread of AAVs from neurons to glia has potential implications in gene therapy. Furthermore, researchers using AAVs to locally modify cells should be aware that they may be modifying astrocytes and neurons at distal areas, which can confound their conclusions. (ii) The experimental platform that we developed overcomes many of the limitations of earlier astrocyte investigations, allowing the unbiased investigation of single astrocytes during behavior and physiological sensory stimulation at the highest spatiotemporal resolution to date. (iii) Vibrissa stimulation only subtly increases the frequency, duration, and size of astrocyte Ca^2+^ MD signals, which can be easily missed in the noise generated by the ongoing activity of the awake brain. These results support both the optimists and critics of neuronal circuit–induced astrocyte Ca^2+^ signaling. (iv) We show stable-over-time (days) Ca^2+^ hotspot maps in single astrocytes that are altered by behavior, suggesting subcellular specialization. These stable subcellular activity hotspots could represent a memory engram.

## MATERIALS AND METHODS

All experimental procedures were approved by the Okinawa Institute of Science and Technology Graduate University (OIST) Institutional Animal Care and Use Committee in an Association for Assessment and Accreditation of Laboratory Animal Care (AAALAC International) accredited facility.

### Animals

Male, 1- to 3-month-old C57/BL6J mice (Japan Clea) were used. Experiments were performed during the dark period of the 12-hour dark/12-hour light cycle.

### Surgeries

Mice were anesthetized by an intraperitoneal injection of a mixture of medetomidine (0.3 mg/kg), midazolam (4 mg/kg), and butorphanol (5 mg/kg) in 0.9% saline solution. Carprofen (5 μg/g, intraperitoneally), dexamethasone (2 μg/g, intramuscularly), and buprenorphine (0.1 μg/g, subcutaneously) were administered to reduce inflammation, immune response, and pain, respectively. Following anesthesia induction, the eyes of the mouse were covered with mycochlorin eye ointment (Sato Pharmaceutical Co. Ltd). The mouse head hair was removed with a hair trimmer and hair removal cream (Veet). The head of the animal was fixed on a stereotactic apparatus, and its skin was sterilized with iodine. If further anesthesia was needed, then 1% isoflurane was administered.

In preparations without chronic window implantation, a single midline sagittal incision of the scalp was performed. The exposed skull was cleaned with the local anesthetic lidocaine. The injection coordinates were based on the Paxinos’ Mouse Brain Atlas (*53*) and previous calibration injections. The skull was thinned at the desired coordinates using a dental drill (OS-40, Osada) with a diamond drill bit (TRUSCO, T2-532M). A sterile glass pipette with a broken tip was used to puncture the thinned skull at the coordinates established to minimize the exposure of the brain. Using beveled glass pipettes (10- to 15-μm tip diameter), AAVs were injected into VPM (1.8 mm posterior, 1.7 mm lateral, and 3.5 mm below the dura) and for some experiments additionally in BX (1.8 mm posterior, 3.2 mm lateral, and 0.5 mm below the dura) for intersectional strategies. AAVs were injected at a rate of ~70 nl/5 min by air pressure application. The pipette was left in place for at least 5 min before and after injection. After injections, the cut skin was closed using superglue. Animals were housed individually and allowed to recover for at least 1 week, unless otherwise stated.

For chronic window implantation surgeries (*54*), the skin was sterilized with iodine before cutting it to expose the skull. The skull was cleaned with lidocaine solution (2% in saline). The region above the injection site in BX was marked, and a circle of ~2-mm radius was drawn around the marking. The skull surrounding the marked circle was thinned with the drill. The bone above the intended craniotomy was gently lifted after gluing a wooden cotton swap with superglue gel to the bone, to expose the dura without damaging it (*55*). Any bleeding was cleaned carefully with carprofen-soaked gelfoam (Pfizer). AAVs were injected into VPM only or both VPM and BX (intersectional strategy) using beveled glass pipettes (10- to 15-μm tip diameter), at VPM: 1.8 mm posterior, 1.7 mm lateral, and 3.5 mm below the dura; and BX: 1.8 mm posterior, 3.2 mm lateral, and 0.5 mm below the dura. In all intersectional strategies, AAVs were first injected in the VPM, followed immediately be a second injection in BX. AAVs were injected at a rate of ~70 nl/5 min by air pressure application. The pipette was left in place for at least 5 min before and after injection. A 5-mm-diameter glass coverslip was placed on the craniotomy and fixed to the bone with super glue. In addition, an aluminum head plate was mounted and fixed with dental cement (Super-Bond). Super-Bond was also used to cover any exposed skull. The animals were individually housed and allowed to recover for at least 1 week.

### Adeno-associated viruses

For an overview of the different strategies for AAV transfer tested in this publication, see table S1.

#### Intersectional strategy—Astrocytes

To assess whether AAV1 tags astrocytes anterograde the injection site, 140 nl (1:1 ratio) of AAV1-CMV-Pl-Cre-rBG (1.2 × 10^13^ GC/ml) and AAV1-hSyn-TurboRFP-WPRE-rBG (3.9 × 10^13^ GC/ml) (University of Pennsylvania Viral Vector Core) was coinjected in VPM, and 100 nl of AAV5-GFaABC1D-Flex-Lck-GCaMP6f-WPRE-SV40 (1.0 × 10^13^ GC/ml) (Sirion Biotech) was injected in BX. For sparse labeling of BX astrocytes with membrane-tagged GCaMP6f for in vivo imaging, we injected 140 nl of AAV1-CMV-Pl-Cre-rBG (1.2 × 10^13^ GC/ml; University of Pennsylvania Viral Vector Core) in VPM and 140 nl of AAV5-GFaABC1D-Flex-Lck-GCaMP6f-WPRE-SV40 (1.0 × 10^13^ GC/ml; Sirion Biotech) in BX.

#### Intersectional strategy—Neurons

To assess whether a similar intersectional strategy can be used to label neurons, 140 nl (1:1 ratio) of AAV1-CMV-Pl-Cre-rBG (1.2 × 10^13^ GC/ml) and AAV1-hSyn-TurboRFP-WPRE-rBG (3.9 × 10^13^ GC/ml) (University of Pennsylvania Viral Vector Core) were coinjected in VPM, and 100 nl of AAV9-Syn-Flex-GCaMP6f-WPRE-SV40 (2.8 × 10^13^ GC/ml) (University of Pennsylvania Viral Vector Core) was injected in BX.

#### Single VPM injection strategies

To test whether injection in VPM alone is sufficient for inducing cell labeling in BX and what elements of the AAV vector are needed to do so, 140 nl (1:1 ratio) of different AAV combinations was injected only in VPM. For sparse labeling of BX astrocytes and neurons along with thalamocortical axons for in vivo imaging, we injected 120 nl (1:1:1 ratio) of AAV1-CMV-Pl-Cre-rBG (1.2 × 10^13^ GC/ml), AAV1-CAG-Flex-GCaMP6f-WPRE-SV40 (1.33 × 10^13^ GC/ml), and AAV1.hSyn-TurboRFP-WPRE-rBG (3.9 × 10^13^ GC/ml) (University of Pennsylvania Viral Vector Core) in VPM.

##### Simple strategy

AAV1-hSyn-TurboRFP-WPRE-rBG and AAV1-CAG-GCaMP6f-WPRE-SV40 (1.33 × 10^13^ GC/ml) (University of Pennsylvania Viral Vector Core) were injected together.

##### Combinatorial strategy

AAV1-CMV-Pl-Cre-rBG and AAV1-CAG-Flex-eGFP-WPRE-bGH (9.16 × 10^13^ GC/ml) (University of Pennsylvania Viral Vector Core) or AAV1-CMV-Pl-Cre-rBG and AAV1-CAG-Flex-tdTomato-WPRE-rBG (4.65 × 10^13^ GC/ml) (University of Pennsylvania Viral Vector Core) were injected together.

### AAV capsid tracking experiment

To track AAV capsids, we used two groups of mice (*n* = 3 each). Each mouse was injected twice, i.e., in each VPM (left and right) once: The first group got the first injection 12 days before the perfusion and the second injection into the contralateral VPM 1 hour before the perfusion. The second group received its first injection 24 hours before the perfusion and the second the injection into the contralateral VPM 1 hour before the perfusion. Injections 1 hour before perfusion were used as positive controls for the anti-VP1 antibody to label AAV capsids at the injection site, within 1 hour after injection. One injection of 140-nl AAV1-CMV-Pl-Cre-rBG and AAV1-CAG-Flex-tdTomato-WPRE-rBG (1:1 ratio) was injected per VPM of each mouse.

#### Brain slice preparation

Mice were deeply anesthetized with a mixture of medetomidine (0.3 mg/kg), midazolam (4 mg/kg), and butorphanol (5 mg/kg) in 0.9% saline solution and transcardially perfused with phosphate-buffered saline (PBS), followed by PLP (4% paraformaldehyde, 1.2% lysine, and 0.2% periodate in 0.1 M phosphate buffer) fixation. The brains were extracted and stored in PLP at 4°C for a minimum of 48 hours. For extended storage, PLP was replaced with PBS. A vibratome (VT1000S, Leica) was used to cut 100-μm-thick coronal slices. The slices were mounted on glass slides with Mowiol and stored at 4°C.

For immunohistochemistry preparation, PLP-fixed brains were cryoprotected using 20% sucrose solution in PBS overnight. The brains were then trimmed and embedded in optimal cutting temperature compound (Tissue-Tek). The samples were frozen at −80°C for 1 hour. A cryotome (Leica, CM3050 S) was used to cut 50-μm-thick coronal slices at −15°C, which were immediately transferred into wells filled with PBS.

#### Immunohistochemistry—AAV capsid tracking

We used immunohistochemistry to tag AAV capsids with anti-VP1 antibody (Antibodies-online, ABIN933221). Brain sections cut with the cryotome were washed in PBS and then incubated in 20% normal goat serum in permeabilization solution (0.3% Triton X-100, 0.05% sodium azide, and PBS) for 1 hour. The serum was replaced with 1:20 mouse anti-AAV1 monoclonal antibody diluted in permeabilization solution. The slices were incubated for 24 hours at 4°C. The samples were washed with PBS and incubated in goat anti-mouse polyclonal secondary antibody Alexa Fluor 488 (Abcam, ab150113) 1:200 (diluted in permeabilization solution) for 2 to 3 hours, in the dark, at room temperature. The brain slices were washed with PBS, mounted on glass slides with DAPI (4′,6-diamidino-2-phenylindole) Antifade Mounting Medium (VECTASHIELD), and stored at 4°C.

#### *Immunohistochemistry—S100*β *and NeuN staining*

We used anti-S100β antibody (rabbit monoclonal, EP1576Y, Abcam, ab52642) as an astrocyte-specific marker. Fifty-micrometer-thick coronal slices (see the “Brain slice preparation” section) were prepared from mice euthanized 3 weeks after intersectional injection of 140 nl (1:1 ratio) of AAV1-CMV-Cre and AAV1-hSyn-TurboRFP in VPM and 100 nl of AAV5-GFaABC1D-FLEx-Lck-GCaMP6f in BX (see the “Intersectional strategy–Astrocytes” section). The brain sections were washed four times in PBS and then incubated in 20% normal donkey serum in permeabilization solution (0.3% Triton X-100, 0.02% sodium azide, and PBS) for 1 hour. The serum was replaced with 1:500 anti-S100β antibody diluted in permeabilization solution. The slices were incubated for 24 hours at 4°C. The samples were washed four times with PBS and incubated with the secondary antibody (donkey anti-rabbit polyclonal antibody, Alexa Fluor 647, Abcam, ab150075) 1:200 (diluted in permeabilization solution) for 2 to 3 hours, in the dark, at room temperature. The brain slices were washed four times with PBS, mounted on glass slides with DAPI Antifade Mounting Medium (VECTASHIELD), and stored at 4°C.

We used anti-NeuN antibody (rabbit monoclonal, EPR12763, Abcam, ab177487) as a neuron-specific marker. Fifty-micrometer-thick coronal slices (see the “Brain slice preparation” section) were prepared from mice euthanized 3 weeks after intersectional injection of 140 nl (1:1 ratio) of AAV1-CMV-Cre and AAV1-hSyn-TurboRFP in VPM and 100 nl of AAV9-Syn-FLEx-GCaMP6f in BX (see the “Intersectional strategy—Neurons” section). The brain sections were washed four times in PBS and then incubated in 20% normal donkey serum in permeabilization solution (0.3% Triton X-100, 0.02% sodium azide, and PBS) for 1 hour. The serum was replaced with 1:1000 anti-NeuN antibody diluted in permeabilization solution. The slices were incubated for 24 hours at 4°C. The samples were washed four times with PBS and incubated with secondary antibody (donkey anti-rabbit, polyclonal antibody, Alexa Fluor 647, Abcam, ab150075) 1:200 (diluted in permeabilization solution) for 2 to 3 hours, in the dark, at room temperature. The brain slices were washed with PBS, mounted on glass slides with DAPI Antifade Mounting Medium (VECTASHIELD), and stored at 4°C.

#### Confocal imaging and analysis in fixed slices

LSM 510 META ConfoCor3 (Carl Zeiss) and LSM 710 (Carl Zeiss) confocal microscopes were used to image fixed brain slices. LSM 510 was used for all ex vivo imaging, except for antibody-labeled capsid samples in the cortex. For LSM 510, a 488-nm argon laser and a 561-nm diode-pumped solid-state (DPSS) laser were used to excite green fluorophores (eGFP, GCaMP6f, Alexa Fluor 488) and red fluorophores (tdTomato, TurboRFP), respectively. A 405-nm diode was used to excite DAPI. Immunostained AAV capsids in BX were imaged with LSM 710 using a 63×/NA 1.46 a-Plan-Apochromat oil objective.

#### Quantification of capsid puncta density in BX

tdTomato^+^ astrocyte and neuron cell bodies (*n* = 15) were randomly selected from three brain slices mainly in L2/3 and L4 (*n* = 3 mice) ~1700 μm lateral of the injection tract. We captured 3D image stacks of 67.5 μm by 67.5 μm field of view (16 bits, 1024 pixels × 1024 pixels), 0.7 μm interspaced from each other at 0.84 AU and averaged eight times. Alexa Fluor 488 and tdTomato were excited using a 488-nm argon laser and a 543-nm helium-neon laser, respectively. For control, 3D stacks with the same parameters were acquired on contralateral hemisphere BX (~1700 μm lateral; ipsilateral the control VPM injection). To quantify the number of capsid puncta, images were first converted to 8 bits, and a 1 × 1 × 1–pixel 3D Gaussian filter was applied. Brightness, contrast, and threshold parameters were established empirically and kept constant. Fluorescent puncta of 0.1 to 0.5 μm^2^ in size were automatically counted using Fiji (*49*) plugin Analyze Particles. The size-range parameter was based on empirical observations of puncta sizes because they could be easily identified by eye. Unpaired two-tailed *t* tests were used to compare the puncta density between (i) BX cell bodies (*n* = 15) versus their local background (*n* = 12) and (ii) BX background versus background of contralateral BX, which was devoid of tdTomato^+^ cell bodies (1 hour after injection of its ipsilateral, control VPM; *n* = 10). The sampling volume was calculated to be equal to the image area × 0.7 μm × number of *Z* sections. Puncta density was calculated to be the number of puncta per sampling volume.

#### Quantification of cell types

A high-resolution confocal microscope LSM 880 Airyscan (Carl Zeiss) was used to image fixed brain slices. A 488-nm argon, 561-nm DPSS, and 633-nm helium-neon lasers were used to excite GCaMP6f, TurboRFP, and Alexa Fluor 647, respectively. A 405-nm diode was used to excite DAPI. Images were taken with a 20× objective.

#### Astrocyte test

We randomly selected the nuclei (DAPI^+^) of BX cells expressing GCaMP6f with a cloud-like morphology in anti-S100β–stained brain slices (*n* = 3 per mouse, three mice). For each cell, we captured a 3D image stack of 142 mm by 142 mm by 7 μm (1024 pixels ×1024 pixels, averaged four times, 1 μm interspaced). We recorded which cells were S100β ^+^ and which ones were not. We reported the percentage of S100β ^+^ and S100β ^−^ cells.

#### Neuron test

We randomly selected a field of view with cell bodies expressing GCaMP6f in BX in anti-NeuN– stained brain slices (*n* = 3 per mouse, three mice). For each slice, we randomly selected a volume of 283 by 283 μm by 25 μm, 1 μm interspaced (1024 pixels × 1024 pixels, averaged four times). In this volume, we counted the GCaMP6f^+^ cell bodies (~6 cells per slice) and identified which cells were NeuN ^+^ and which ones were not. We reported the percentage of NeuN ^+^and NeuN ^−^ cells.

#### Quantification of astrocyte and neuron density in BX

We used brain slices from mice injected using intersectional strategies to label astrocytes (see the “Intersectional strategy—Astrocytes” section, *n* = 3 slices per mouse, four mice) or neurons (see the “Intersectional strategy—Neurons” section, *n* = 3 slices per mouse, three mice). We used confocal-tile scanning to image GCaMP6f^+^ cells in the BX. We manually counted all the cell bodies in the BX column with the most GCaMP6f^+^ cells using Zen Lite software (Zeiss). We divided the number of cells by the volume of each column to estimate the density of astrocytes and neurons. Furthermore, we divided each column into L1, L2/3, L4, and L5/6 based on (*56*) (~total width: 1125 μm, L1: 37 μm, L2/3: 234 μm, L4: 259 μm, and L5/6: 595 μm, scaled). We divided the number of labeled cells by the volume of each layer to estimate the density of astrocytes and neurons in each preparation, respectively. The characteristic barrel-like projection patterns of TurboRFP^+^ thalamocortical neurons was used as assistance in delineating cortical columns and layers.

### 2P imaging in behaving animals

One week after chronic cranial window implantation, mice were habituated by periodic handling (~20 min daily for ~1 week) and exposure to head fixation on a cylindrical treadmill (*r* = 7.4 cm). A vibrissa stimulus was introduced by inserting a wooden rod (toothpick segment) in the treadmill to intercept (one stimulus per rotation, equaling one stimulus every 46.5 cm) the vibrissae contralateral to the injection site while the animal ran. Animals were briefly anesthetized with 2% isoflurane before head fixation and left to rest and recover from anesthesia for ~10 min before 2P imaging. In vivo 2P microscopy was used to image Ca^2+^ signaling ~3 weeks after injection (depending on the expression of GCaMP6f). A charge-coupled device (CCD) camera and infrared light source (920 nm, Thorlabs) were used to monitor behavioral activity (focused on the vibrissa pad of the animal) and vibrissa stimulation by the wooden rod. A rotary encoder (E6A2, Omron) was used to record locomotion.

In vivo imaging was performed using a custom-built combined wide field and 2P microscope (MOM, Sutter Instrument) with a 25×/NA 1.05 water immersion objective with a 2-mm working distance (Olympus). A Ti:sapphire femtosecond pulsed laser (Vision II, Coherent) was used to excite fluorescence at 950 nm (power measured after the objective <60 mW). The back aperture of the objective was underfilled to elongate the PSF of excitation to ~4 to 5 μm in axial direction and 1 μm in the imaging plane (*39*). A resonant scanner was used to acquire images at 30.9 Hz. Fluorescence was detected by two GaAsP photomultiplier tubes (Hamamatsu) in the spectral range of 490 to 560 nm (green) and 570 to 640 nm (red) separated by a 565-nm dichroic mirror (Chroma). Commercial software (MScan, Sutter Instrument) controlled the microscope, analog channels, and CCD camera. Imaging was done ~300 μm below the dura (L2/3) in BX with 512 × 512 pixels and 94 μm by 94 μm field of view. To repeatedly image the same astrocyte, a 5×/NA 0.25 air objective (Zeiss) was used, followed by the 25× objective to map the blood vessel pattern on the brain surface directly above the astrocyte of interest. To find the same z-plane over consecutive days (1 to 3 days after the first recording), we used depth information, autofluorescent puncta patterns, the morphology of the astrocyte, and the blood vessel pattern passing through the astrocyte.

#### Multiple-pole experiment

Three more mice were used by a different experimenter to investigate the effect of high-frequency stimulation on astrocyte Ca^2+^ signaling. Mice were habituates (see above) on a treadmill with 23 poles equally interspaced from each other (2 cm) along the circumference stimulating the vibrissae contralateral of the imaging site. One astrocyte per mouse was recorded as before, two times per day across two consecutive days (*n* = 4 recordings per astrocyte, 12 recordings total). During the imaging, mice were free to run on the treadmill with either 1 or 23 poles. After the first recording (~1 hour long), poles were added or subtracted accordingly, and the same astrocyte was recorded for ~1 more hour. Event characteristics (frequency, amplitude, duration, and size) were collected during these recordings. We compared the events collected during (i) locomotion without pole interaction with vibrissa, (ii) locomotion with 1-pole interaction (within 1.5 s of interaction), and (iii) locomotion on 23-pole treadmill. We divided the mean event characteristics obtained during each state by the respective mean event characteristics obtained during the respective recording while the animal was at rest. We performed this normalization to minimize interrecording variability of baseline activity. A notable difference was a cutoff frequency of 3 Hz used to compensate for lower signal-to-noise recording to minimize bleaching and phototoxicity. Recordings (30.9 Hz) were averaged nine times instead of three times like other experiments to sufficiently increase the sound-to-noise ratio to ensure correct event detection by AQuA.

### In vivo data analysis

#### Behavioral data extraction

A CCD camera was used to detect vibrissa stimuli. The sharp change in infrared light intensity detected by the camera at the region of the vibrissa pad contralateral to the injection side indicated the transit. The vibrissa pad region was manually selected in Fiji (*49*). A threshold light intensity binarized the vibrissa stimulation events (0 = no vibrissa interception and 1 = vibrissa interception).

The behavioral state of the animal was determined as a binary signal: running or resting. Movement of the mouse on the treadmill was extracted from the analog signal of the rotary encoder. If the running mouse stopped for less than 1.5 s and then continued running, then its state was considered continuous running. If the mouse stopped for more than 1.5 s, then its state was defined as at rest. If the wooden rod intercepted the vibrissae, then it was defined as a vibrissa stimulus. In most cases, the animal was running when the wooden rod intercepted its vibrissae, defined as vibrissa stimulation. Sometimes, the animal would stop and explore the wooden rod with its vibrissae during rest, defined as vibrissa exploration.

#### Preprocessing

2P microscopy movies (30.9 Hz), rotary encoder analog signals, and animal behavior movies were synchronously recorded using MScan software. Proprietary video format files (MDF, Sutter Instrument) were converted to Tiff using commercial software (MView, Sutter Instrument). Time-lapse recordings were preprocessed using Fiji (*49*). Movement artifacts in the imaging plane was corrected using the TurboReg (*50*) plugin (batch mode, translation mapping) and a custom macro to automate the process. Videos were then visually inspected to confirm movement artifact correction. A 3D Gaussian filter with sigma = 1 pixel in *x*, *y*, and *t* was applied to all 2P movie stacks using Fiji. The 30.9-Hz raw video was binned to 10.3 Hz.

#### AQuA processing

AQuA (*23*) was used for unbiased identification and characterization of astrocyte Ca^2+^ MD signals. AQuA applies machine learning techniques to model Ca^2+^ events in an event-based, data-driven way that does not impose a priori assumptions about the data. All preprocessed time-lapse movies used for AQuA processing had a nominal spatial resolution of ~0.18 μm per pixel (optical resolution based on the PSF of the microscope: 1.0 μm; therefore, fivefold oversampling) and a temporal resolution of 10.3 Hz. AQuA performed further preprocessing by applying a Gaussian filter (smoXY = 2 SD). AQuA estimated the SD of the noise (sigma). A conservative Δ*F/F* threshold was set for signal detection equal to four times sigma (thrARScl = 4). In addition, events with lower Δ*F/F* than 20% of peak *F* were discarded (minShow1 = 0.2). AQuA excluded any Ca^2+^ signals composed of less than six pixels (minSize = 6 pixels) and less than 0.2 s in duration (seedRemoveNeib = 2). Events smaller than 2 μm^2^ (2× PSF) were also subsequently filtered out. No pixels were removed close to the imaging boundary (regMaskGap = 0). A temporal threshold (thrTWScl = 2) was set to discriminate between several events occurring in the same spatial location (defined as delta = thrTWScl × sigma). Voxels (*x*, *y*, and *t*) with higher values compared to their spatial and temporal neighbors are termed seeds. To determine whether neighboring pixels to each seed are similar enough to be included in the signal, a growing z-threshold relating to noise is used (thrExtZ = 2). The assigned pixels to seeds form super voxels that, in turn, can be combined to form super events. This is classified by AQuA using three parameters: rising time uncertainty (cRise = 2), slowest delay propagation (cDelay = 2), and propagation smoothness (gtwSmo = 1). A *z*-score threshold was set to distinguish events from noise (zThr = 2). AQuA also compensates for possible bleaching that can occur during long recordings. This is done by cutting the video into substacks (cut = 200 frames) to calculate baseline fluorescence (F0) through a moving average filter (movAvgWin = 25 frames). Unmentioned parameters were set to default. The same parameters were used for all single-astrocyte Ca^2+^ MD signal analysis.

AQuA was used to extract four event characteristics: size, amplitude, duration, and frequency of events. Size refers to the maximum spatial extent (or spread) of the Ca^2+^ signal (in square micrometers), amplitude refers to the maximum Δ*F/F* of the signal, duration refers to the maximum temporal extent of the signal (in seconds), and frequency refers to the number of events detected over time. Each event is described as a 2D binary footprint, whose area (basic.area) represents the event size (in square micrometers). The peak Δ*F/F* of each event (curve.dffMax2) was used as amplitude (Δ*F/F*). Duration was calculated as the time between the starting (loc.t0) and stopping frames (loc.t1) of each event.

#### Statistics

Statistics were performed using Python (3.7). The normality of the distributions was assessed using the Shapiro-Wilk test for data <50 data points or Kolmogorov-Smirnov test for data with >50 data points. If the *P* value was less than 0.05 (chosen alpha level), then we rejected the null hypothesis and considered the data tested not to be normally distributed. A straight line of the quantile-quantile plot was also used as evidence of a normal distribution. Normally distributed data was analyzed using unpaired *t* test (two independent groups), paired *t* test (two paired groups), one-way analysis of variance (ANOVA) (multiple independent group comparison) or one-way repeated-measures ANOVA (multiple groups, within group comparison). Nonparametric data were analyzed using Kruskal-Wallis test (multiple independent groups). To counteract the problem of multiple comparisons, we used Tukey’s post hoc test for normally distributed data. For nonnormal distributed data, we used the more conservative Bonferroni correction. Data were plotted as means ± 95% CI. Only two-tailed tests were used, and the significance threshold was set to *P* < 0.05.

Data fitting was also performed in Python. Skewed normal probability density functions (scripy.stat.skewnorm) were fitted to the data and characterized on the basis of (*51*). To plot and compare complementary cumulative distribution functions, we used the “powerlaw” Python package (*57*). We visualized and compared power law, exponential, and lognormal fits to the empirical event-size data. For this fitting, we set a minimum Ca^2+^ event size threshold of 5 μm^2^.

#### Astrocyte Ca^2+^ MD signal analysis

For the analysis of Ca^2+^ MD signals, we used six astrocytes labeled with membrane-tagged GCaMP6f. Two of these astrocytes were imaged a second time 1 day later, and another one 3 days later. For the analysis, all nine recordings were used unless otherwise stated. Mice transitioned between rest and run states during all recordings. During three of nine recordings, mice did not stop to explore the vibrissa stimulus. Therefore, no data were gathered during vibrissa exploration state for these astrocytes. Four Ca^2+^ event characteristics were analyzed: amplitude (Δ*F/F*), duration (s), size (μm^2^), and frequency (number of events/*t*, *t* = time in seconds or minutes) during the four behavioral states: rest, run, vibrissa stimulation, and vibrissa exploration. We compared the means of event characteristics (between group analysis) for all events (*n* = 50,787) from all astrocyte recordings (*n* = 9) during rest (*n* = 29,637), run (*n* = 15,123), vibrissa stimulation (*n* = 2906), and vibrissa exploration (*n* = 3121). Event characteristics were grouped and averaged per astrocyte recording to control for variability between astrocyte recordings and recording time. We compared the mean event characteristics of all recordings (within group design, paired *t* test). All events (*n* = 50,787) from all astrocyte recordings (*n* = 9) were used to investigate the probability distributions of event characteristics (amplitude, duration, and size) during different states. The continuous recoding times (*n* = 9) were 22, 35, 40, 40, 75, 75, 79, 79, and 106 min long.

To compare event characteristics during state transitions, all recordings were aligned to a transition point. The transition points were rest-to-run (226 transitions: 9-s intervals and 4263 Ca^2+^ events), run-to-rest (177 transitions: 9-s intervals and 3089 Ca^2+^ events), and vibrissa stimulation during run (279 transitions: 6-s intervals and 5333 Ca^2+^ events). Transition events were only selected if the behavioral state was stable during the pre- and posttransition period. For example, during a rest-to-run transition, the animal was at rest for at least 3 s, followed by at least 6 s of continuous running. The onset time (loc.t0) of each event was used to determine the Ca^2+^ MD signal characteristics in relation to the time of state transition.

#### Dual axon-astrocyte Ca^2+^ signal analysis

By coinjecting AAV1-CMV-Cre, AAV1-CAG-Flex-GCaMP6f, and AAV1-hSyn-TurboRFP (see the “Adeno-associated viruses” section) in the VPM, we labeled BX astrocytes with GCaMP6f and thalamocortical axons (VPM to BX) with GCaMP6f and TurboRFP (*n* = 3 mice, three astrocytes). Because of sparse astrocyte labeling, some axon boutons (identified as functional puncta expressing both GCaMP6f and TurboRFP) were found inside the astrocyte territory and some outside (see fig. S4B). We used the Ca^2+^ activity of axon boutons outside the astrocyte territory as controls to confirm thalamocortical neuron activity indeed increases with vibrissa stimulation and locomotion as expected.

The Ca^2+^ activity of axon bouton signals outside the gliapil during behavioral-state transitions was extracted with AQUA. AQUA parameters were optimized for extracting axon activity. The minimum event size cutoff threshold was set to >0.74 μm (minSize = 4), the spatial smoothing level was set to 1 SD (SmoXY = 1), and the active threshold scale was set to 6 SD of noise (thrARScl = 6). In addition, 50 pixels close to the image border (regMaskGap = 50) were removed. Signals represent distinct points of [Ca^2+^]_i_ elevation, not distinct axons. We investigated changes in mean frequency (number of events/s) of axon Ca^2+^ signals during behavioral-state transitions like we did previously with astrocytes. The behavioral-state transitions investigated and their respective axon Ca^2+^ signals were rest to run (174 intervals and 8613 events), run to rest (52 intervals and 2274 events) and whisker stimulation during run (756 intervals and 28,232 events). Using this preparation (GCaMP6f not membrane-tagged, no AAV injection in BX), we also retested whether astrocytes responded with [Ca^2+^]_i_ elevation to whisker stimulation (756 intervals and 6091 events). We selected astrocyte processes (inside gliapil) constrained within manually drawn ROIs (*n* = 35 from three astrocytes) devoid of TurboRFP-labeled axon boutons to minimize contamination from axon activity. We tested again how the mean frequency (number of events/s) of Ca^2+^ signals initiated within these ROIs changed during whisker stimulation (756 intervals and 6091 events).

#### Astrocyte activity heatmaps

Astrocyte activity maps refer to overall activity patterns observed in astrocytes during long recording times (typically tens of minutes) represented in the form of a heatmap. A heatmap represents the proportion of events in space detected by summing the 2D event footprints detected by AQuA in time.

#### Heatmap generation

Motion-corrected, 2P Ca^2+^ imaging data of single astrocytes were processed by AQuA. The Ca^2+^ signals were summarized as 2D footprints and binarized to pixel value = 1. All binarized events were summed in time while maintaining their spatial coordinates to develop activity heatmaps for each astrocyte recording. Some heatmaps were limited to Ca^2+^ events occurring during specific behavioral states (rest, run, vibrissa stimulation, vibrissa exploration, and all states). In these cases, only frames associated with the respective behavioral state were considered. Heatmaps were normalized to 1 min and to their respective maximum pixel value unless otherwise noted.

#### Simulation of random distributions

Simulations of activity heatmaps were created by randomly distributing binarized Ca^2+^ signals onto a mask corresponding to the real, recorded astrocyte. Ca^2+^ signals were approximated as ellipses of random orientation, center, and eccentricity. These ellipses were incrementally added within the bounds of the astrocyte previously imaged (binarized maximum projection mask). The area of each ellipse was randomly sampled from the list of event areas corresponding to the respective, real astrocyte recording. The total area of all ellipses in each random simulation was equal to the total area of real Ca^2+^ events of their respective astrocyte recording. For example, if the total area of ellipses added to the simulation was equal to the total area of real-event 2D footprints, then no more ellipses were added.

#### Heatmap comparison

Heatmap hotspot patterns were compared using the total continuous recording, video segments corresponding to the state of the animal, or subsequences of the respective video recording (sequences) for within-day or between-day comparisons.

##### Within day

To compare activity heatmaps within same-day recordings and between their respective simulations (random distributions), 2D image cross correlation (512 × 512 pixels; Pearson cross correlation) was used. One of the images was translated to a position (translation vector) of maximum correlation. The maximum PCC was used as the correlation metric between the images.

##### Between days

To correlate activity heatmaps between days, we created an activity mask to establish the boundaries of cell activity throughout the whole recording. The mask array had binary *x* and *y* values, where 1 represents an event taking place. 2D cross correlation was used to find the maximum correlation between a stationary sample mask and one translated by a move vector. This allowed us to align the masks of the sample pairs and use 2D correlation between the heatmap pairs.

#### Sequences

Sections of continuous video recordings are referred to as sequences. 2D correlation between heatmaps generated from 70-min-long recordings and their shorter subsequences (*t* = 2, 5, 10, 15, 25, 30, 35, and 70 min) were used to determine the approximate recording time needed to capture a reliable Ca^2+^ activity map. Seventy-minute movies were created by omitting frames after 70 min in five recordings longer than 70 min.

Similarly, 2D correlation between the activity heatmaps of rest state sequences of the same astrocyte (within day) were used to determine the stability of heatmaps over individual recordings. The sequences were created from five astrocyte movies, 70 min each. Each video was split into three subsequences of equal duration corresponding to the rest state of the animal. Their respective heatmaps were 2D-correlated to each other (three subsequences per astrocyte correlated, five astrocytes, 15 correlations). Alternatively, subsequences were correlated with the total running activity heatmap of their respective astrocyte (state comparison, three correlations per astrocyte, five astrocytes). Between-day correlation of subsequence heatmaps (*n* = 12) was obtained from the same astrocytes (*n* = 2) but during different day recordings (days 0 and 1, 18 correlations between days).

## Notes

View/request a protocol for this paper from *Bio-protocol*.
