# Supplementary material for: Ca+ activity maps of astrocytes tagged by axoastrocytic AAV transfer
Source: Sci Adv. 2022 Feb 9;8(6):eabe5371. doi: 10.1126/sciadv.abe5371 (PMC8827655; doi:10.1126/sciadv.abe5371)
Supplement: Supplementary file 1 — Figs. S1 to S10 Table S1 Legends for movies S1 to S3 [file sciadv.abe5371_sm.pdf]

Science Advances
AAAS

Supplementary Materials for

Ca^{+} activity maps of astrocytes tagged by axoastrocytic AAV transfer

Leonidas Georgiou*, Anaí Echeverría, Achilleas Georgiou, Bernd Kuhn*

*Corresponding author. Email: leonidas.g88@gmail.com (L.G.); bkuhn@oist.jp (B.K.)

Published 9 February 2022, *Sci. Adv.* **8**, eabe5371 (2022)
DOI: 10.1126/sciadv.abe5371

The PDF file includes:

Figs. S1 to S10
Table S1
Legends for movies S1 to S3

Other Supplementary Material for this manuscript includes the following:

Movies S1 to S3

Supplementary Figures

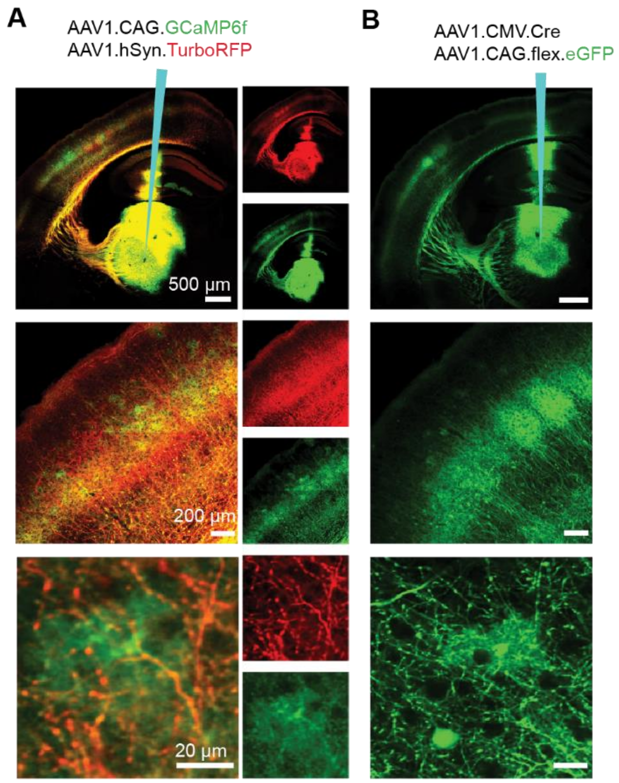

Fig. S1. Barrel cortex astrocytes and neurons can be labelled with a single AAV injection in VPM.

(A) AAV1-hSyn-TurboRFP and AAV1-CAG-GCaMP6f injected in VPM label neurons and astrocytes in BX with GCaMP6f. Thalamocortical projections are double labeled with GCaMP6f and TurboRFP. This indicates that intersectional approaches or Cre-recombinase are not required for AAV mediated anterograde transduction of BX astrocytes and neurons. Note: AAV1-hSyn-TurboRFP will also transfer to BX neurons, however the expression level under the hSyn promoter is too low to be visible here.

(B) AAV1-CMV-Cre and AAV1-CAG-Flex-eGFP injected in VPM labels neurons and astrocytes in BX with eGFP. This indicates that intersectional approaches and/or a specific fluorescent protein (i.e., GCaMP6f) are not required for AAV mediated anterograde transduction of astrocytes and neurons.

Confocal images were taken from fixed brain slices of mice sacrificed two weeks after injections.

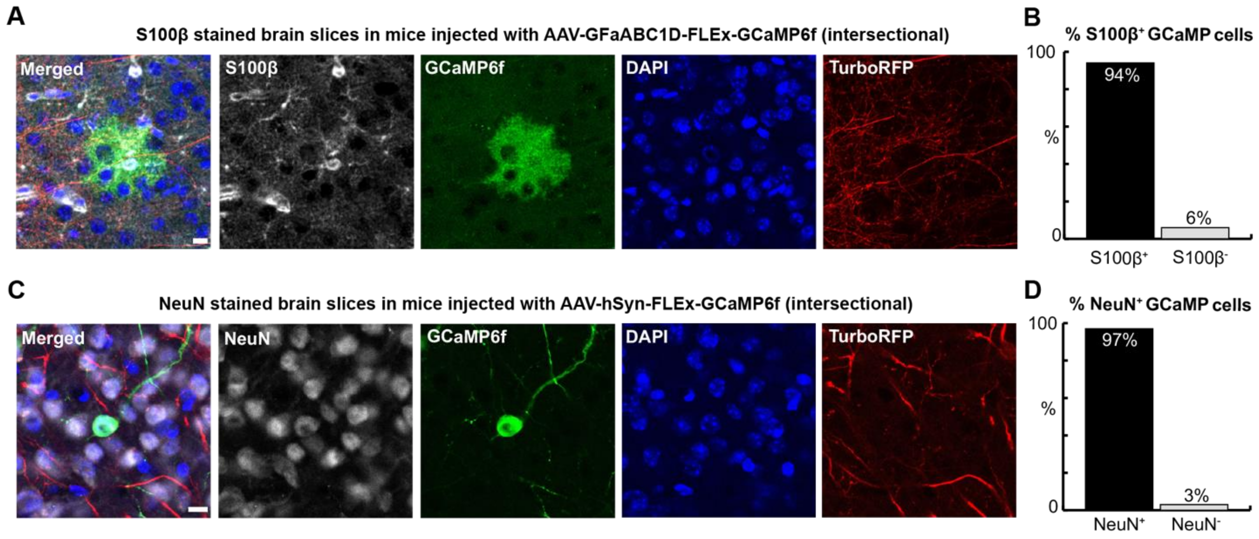

Fig. S2. Cell type specific antibody labelling of astrocytes and neurons

(A) Barrel cortex cell (green) and thalamocortical axons (red) in brain slice stained with anti-S100β antibody (gray) and DAPI (blue), 3 weeks after injection of AAV1-CMV-Cre and AAV1-hSyn-TurboRFP in VPM, and AAV5-GFaABC1D-FLEx-lck-GCaMP6f in BX. Scale bar = 10 µm.

(B) Percentage of GCaMP6f labelled cells in BX that are S100β positive (black bar) and S100β negative (gray bar) in mice injected as in (A) (n = 32 cells, 12 slices, 3 mice).

(C) Barrel cortex cell (green) and thalamocortical axons (red) in brain slice stained with anti-NeuN antibody (gray) and DAPI (blue), 3 weeks after injection of AAV1-CMV-Cre and AAV1-hSyn-TurboRFP in VPM, and AAV9-hSyn-FLEx-GCaMP6f in BX. Scale bar = 10µm

(D) Percentage of GCaMP6f labelled cells in BX that are NeuN positive (black bar) and NeuN negative (gray bar) in mice injected as in (C) (n = 70 cells, 9 slices, 3 mice).

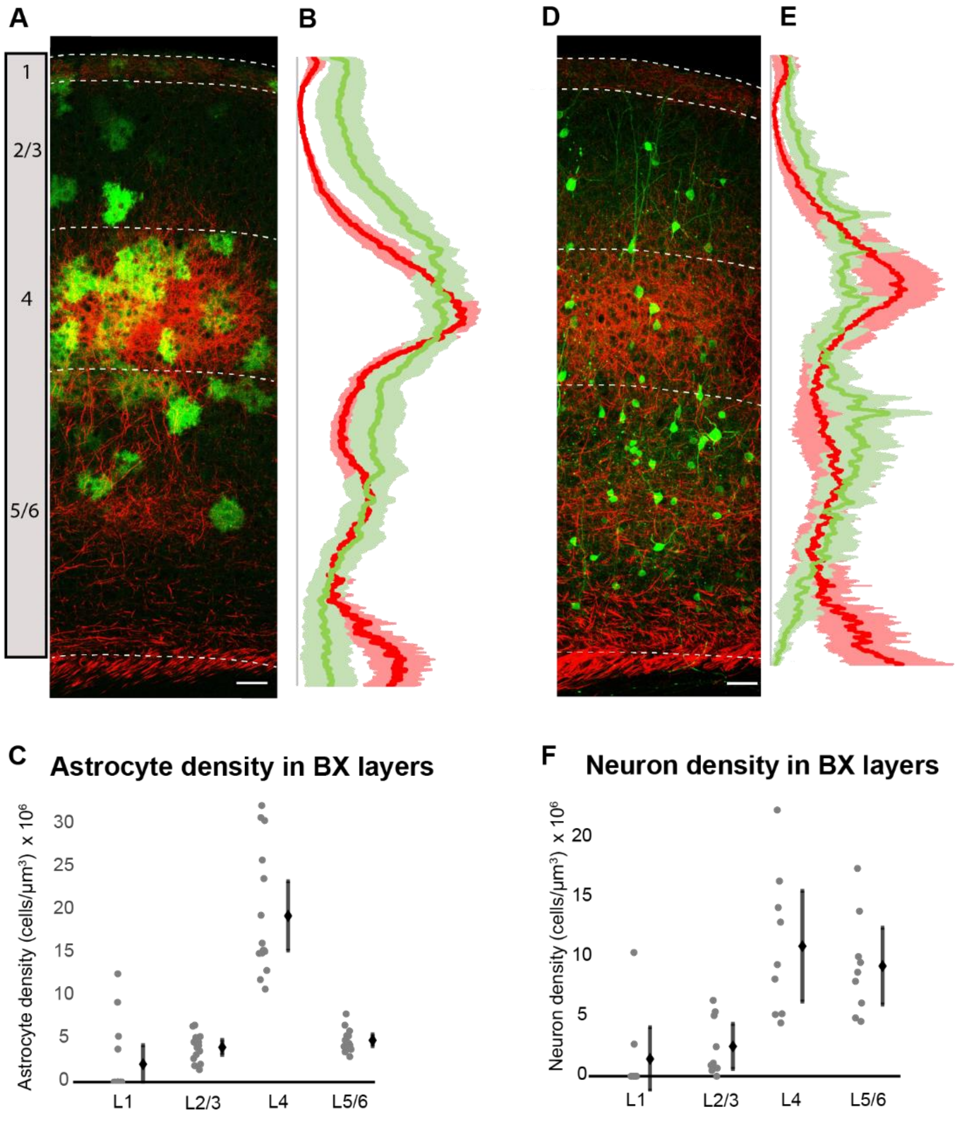

Fig. S3: Distribution of astrocytes and neurons along barrel cortex layers

Confocal imaging of coronal cortical slices of mice sacrificed 3 weeks after intersectional injection of AAV1-CMV-Cre and AAV1-hSyn-TurboRFP in VPM and **(A-C)** AAV5-GFaABC1D-FLEx-Lck-GCaMP6f or **(D-F)** AAV9-Syn-FLEx-GCaMP6f in barrel cortex (BX).

(A) Astrocytes (green) and thalamocortical axons (red) distributed in the six cortical layers (L1, L2/3, L4, L5/6). Scale bar = 50 µm.

(B) Normalized fluorescence intensity ± CI distribution of TurboRFP labelled thalamocortical axons (red) and GCaMP6f labelled astrocytes (green) along the layers of single barrels (n = 3 mice, 12 slices).

(C) Mean density ± CI of astrocytes in L1, L2/3, L4 and L5/6 of the BX.

(D) Neurons (green) and thalamocortical axons (red) distributed in the six cortical layers in

BX. Scale bar 50 μm.

(E) Normalized fluorescence intensity ± CI distribution of TurboRFP labelled thalamocortical axons (red) and GCaMP6f labelled neurons (green) along the layers of single barrels (n = 3 mice, 9 slices).

(F) Mean density ± CI of neurons in L1, L2/3, L4 and L5/6 of the BX.

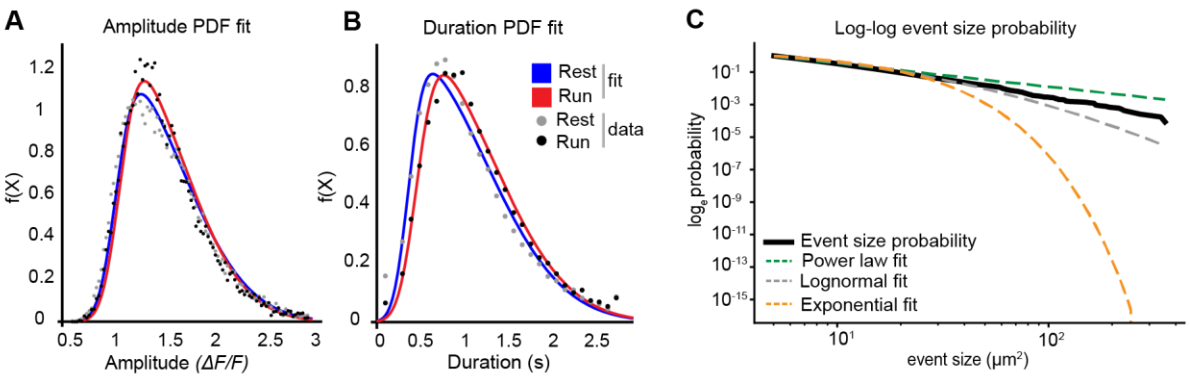

Fig. S4. Ca^{2+} event characteristics: distributions and fitting.

(A) Probability density f(X) of event amplitudes (*ΔF/F*) during rest (gray dots) and run (black dots) states, fit by skewed distribution functions, corresponding to rest (blue line) and run (red line, black dots) states.

(B) Probability density (f(X)) of event durations (s) during rest (gray dots) and run (black dots) states, fit by skewed distribution functions, corresponding to rest (blue line) and run (red line) states.

(C) Complementary cumulative distribution function of event size probability (*black)* fitted by power law (green, $\alpha = 2.28$, $\sigma = 9 \times 10^{-3}$), exponential (orange), and lognormal (gray, $\mu = 1.29$, $\sigma = 1.06$) functions (57).

Number of recordings = 9, number of mice = 3, number of astrocytes = 6.

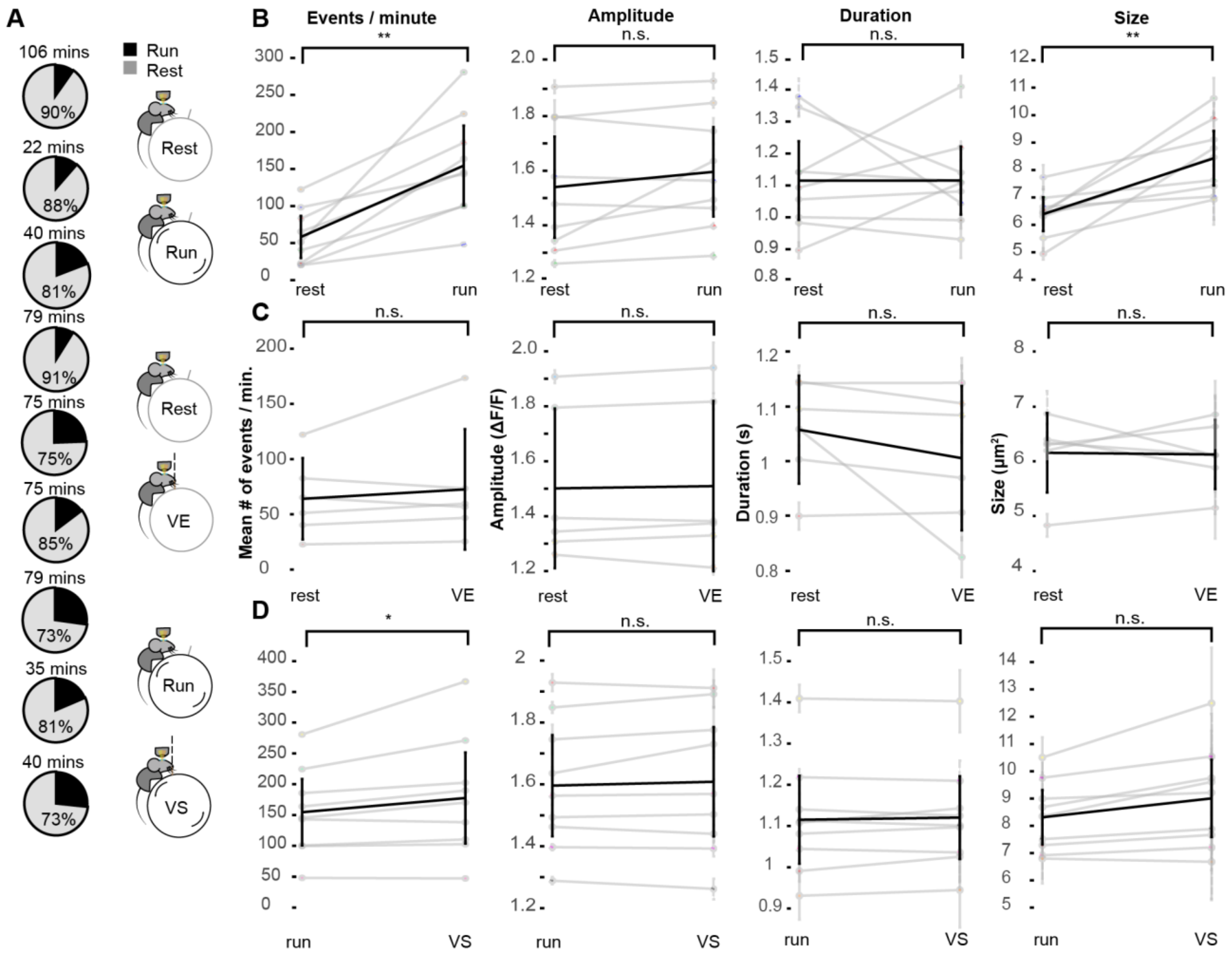

Fig. S5. Astrocyte Ca^{2+} event characteristics change with the state of the animal.

(A) Total imaging time and ratio of total rest and run time for the 9 astrocyte recordings analyzed.

(B) Mean frequency of events per minute, amplitude (*ΔF/F*), duration (*s*), and size (*μm^2*) changes between rest and run.

(C) Mean frequency of events per minute, amplitude (*ΔF/F*), duration (*s*), and size (*μm^2*) changes between rest and vibrissa exploration (VE).

(D) Mean frequency of events per minute, amplitude (*ΔF/F*), duration (*s*), and size (*μm^2*) changes between run and vibrissa stimulation (VS).

Individual astrocyte recordings (gray) and their mean ± CI (black line). Paired, two tailed t-test, $p < 0.01$ **, $p < 0.05$ *, $p > 0.05$ non-significant (n.s.).

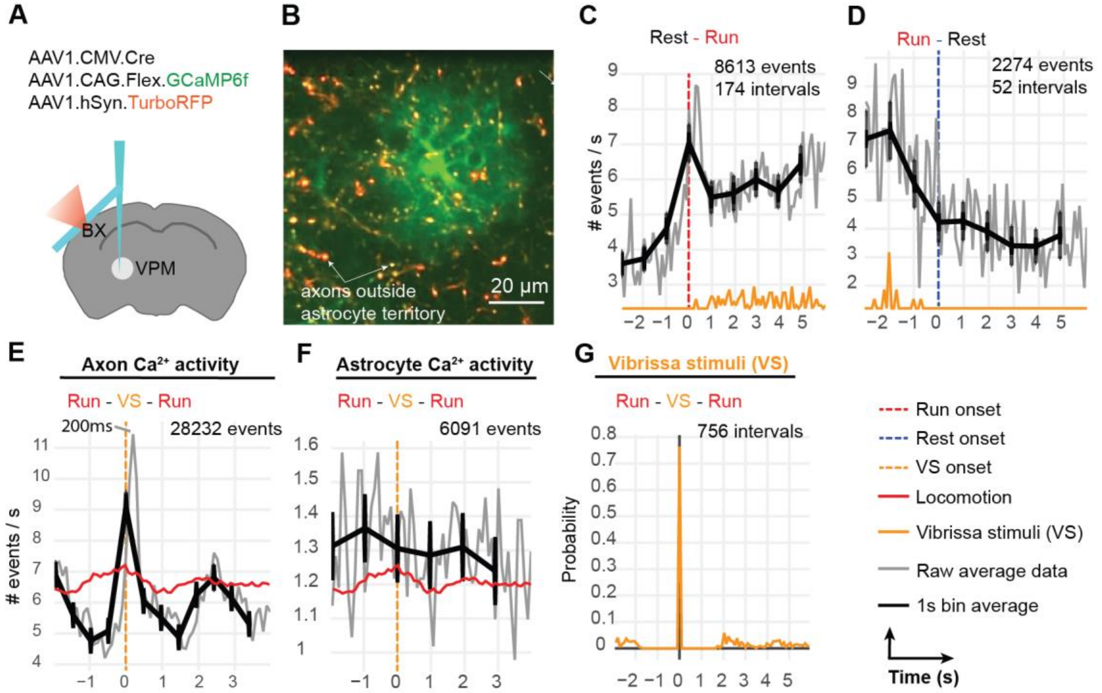

Fig. S6. Simultaneous imaging of axonal and astrocytic Ca^{2+} MD activity after axo-astrocytic AAV transfer.

(A) AAV injection strategy to study axon - astrocyte interactions in BX with two-photon microscopy.

(B) BX L2/3 astrocyte labelled with GCaMP6f (green) and thalamocortical axons labelled with both GCaMP6f (green) and TurboRFP (red).

(C) Mean number of axonal Ca^{2+} events/second during rest to run transitions (8613 events, 174 intervals).

(D) Mean number of axonal Ca^{2+} events/second during run to rest transitions (2274 events, 52 intervals)

(E) Mean number of axonal Ca^{2+} events/second 2s before and 4s after the onset of vibrissa stimulation (orange line) (28232 events, 756 intervals).

(F) Mean number of astrocytic Ca^{2+} signals per second (area of GCaMP6f-labeled axon boutons excluded) (n = 35) 2s before and 4s after the onset of vibrissa stimulation (orange line) (6091 events, 756 intervals).

(G) Probability distribution of vibrissa stimuli during vibrissa stimulation trials (756 intervals). Axonal activity was restricted to axons outside the astrocyte territory. Data represented as ± 95% confidence interval.

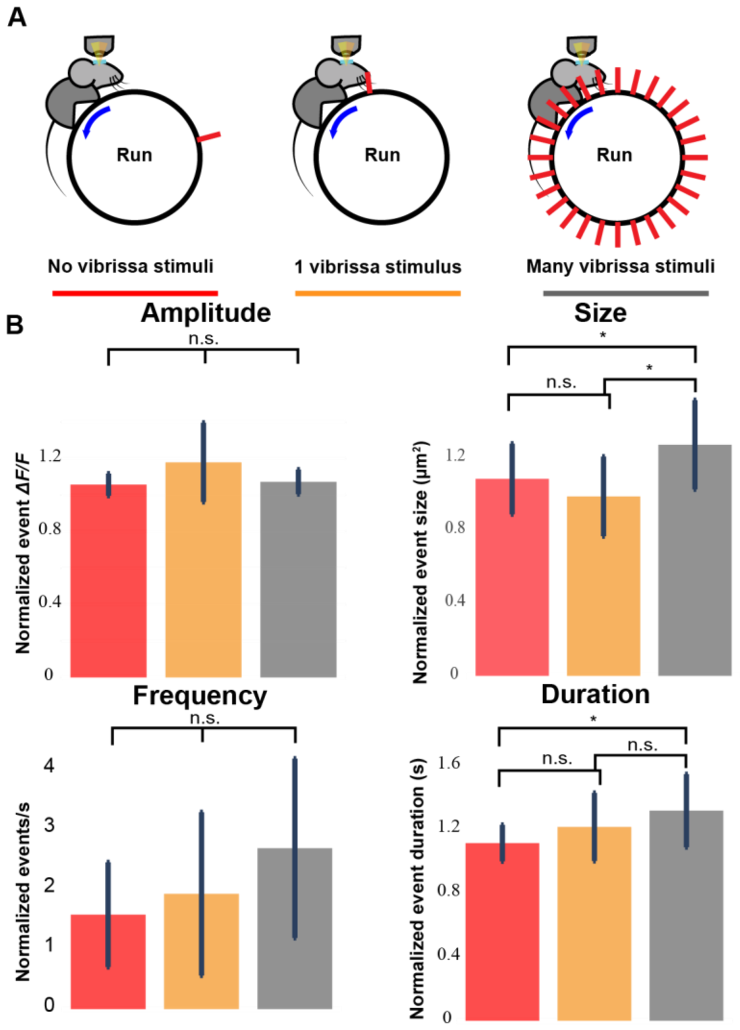

Fig. S7. High frequency vibrissa stimulation subtly increases astrocyte Ca^{2+} event characteristics

(A) Astrocyte Ca^{2+} events were investigated during locomotion (Run) without vibrissa stimulation (red), single vibrissa stimulation (orange), or multiple (23-pole), interspaced vibrissa stimuli (gray).

(B) Mean normalized, event amplitude ($\Delta F/F$), size (μm^2), frequency (events/s) and duration (s) changes during no vibrissa stimulation (red), single vibrissa stimulation (orange) and high frequency vibrissa stimulation (gray).

12 recordings, 3 mice, 3 astrocytes. Each astrocyte was recorded 4 times: Vibrissa stimulation with 1 pole and 23 poles, on 2 consecutive days.

Mean ± CI (black line). One-way repeated measures ANOVA, $p < 0.05$ *, $p > 0.05$ non-significant (n.s.). Data was normalized to the mean Ca^{2+} activity (respective event characteristic) of the same recording during rest state of the animal. Cutoff frequency = 3Hz.

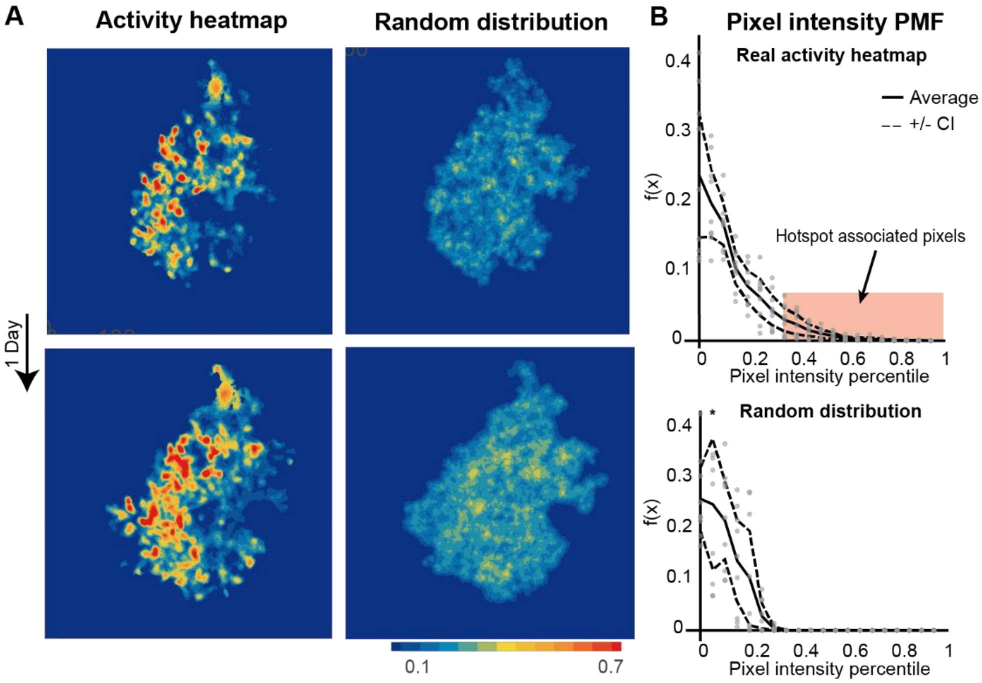

Fig. S8. Ca^{2+} hotspots are not randomly distributed

(A) Natural, astrocyte activity heatmap (all states) during day 0 (top left) and day 1 (bottom left) compared to their respective simulated heatmaps generated by random distribution of events (right). All heatmaps are saturated to their 70% maximum pixel value. Simulated heatmaps are normalized to the same range of pixel values as their respective natural heatmap.

(B) Probability mass functions of normalized pixel intensity values in natural (Real activity heatmap, top) and simulated (Random distribution, bottom) heatmaps. Mean frequency of pixel intensity values (normalized to 1) of 9 astrocyte recordings (mean ± CI). Pixels with higher intensity values than expected of random distributions (within red box) are associated with hotspot activity.

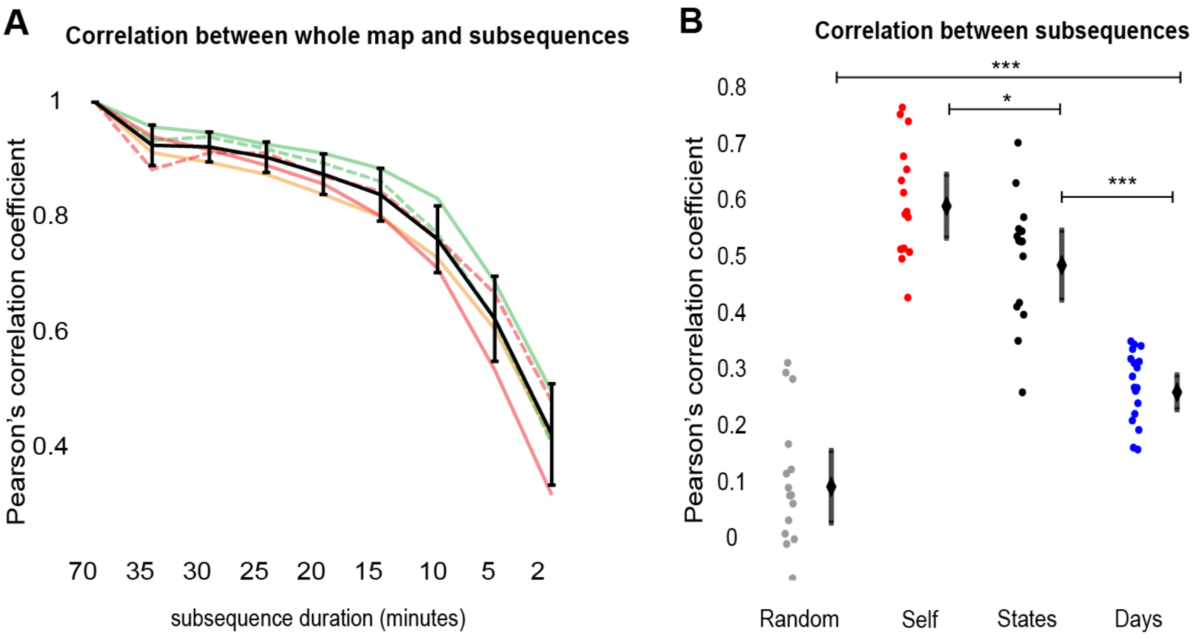

Fig. S9. Heatmap correlations

(A) Pearson's correlation coefficient between the full activity heatmaps of 70-min-long astrocyte recordings (n = 5, 70 min each, colored lines) and their respective subsequences of different duration. Black line = mean ± CI.

(B) Pearson's correlation coefficient between rest state subsequences (3 per astrocyte, 5 astrocytes) vs: their respective simulated heatmaps (gray), other subsequences of same day recordings during rest state (red), run state heatmaps of same day recordings (black), and subsequences derived from the same astrocyte, recorded a day later, corresponding to rest state (blue). Data represented as mean ± CI. Compared using one-way ANOVA with Tukey's HSD, $p < 0.05$ *, $p < 0.001$ ***

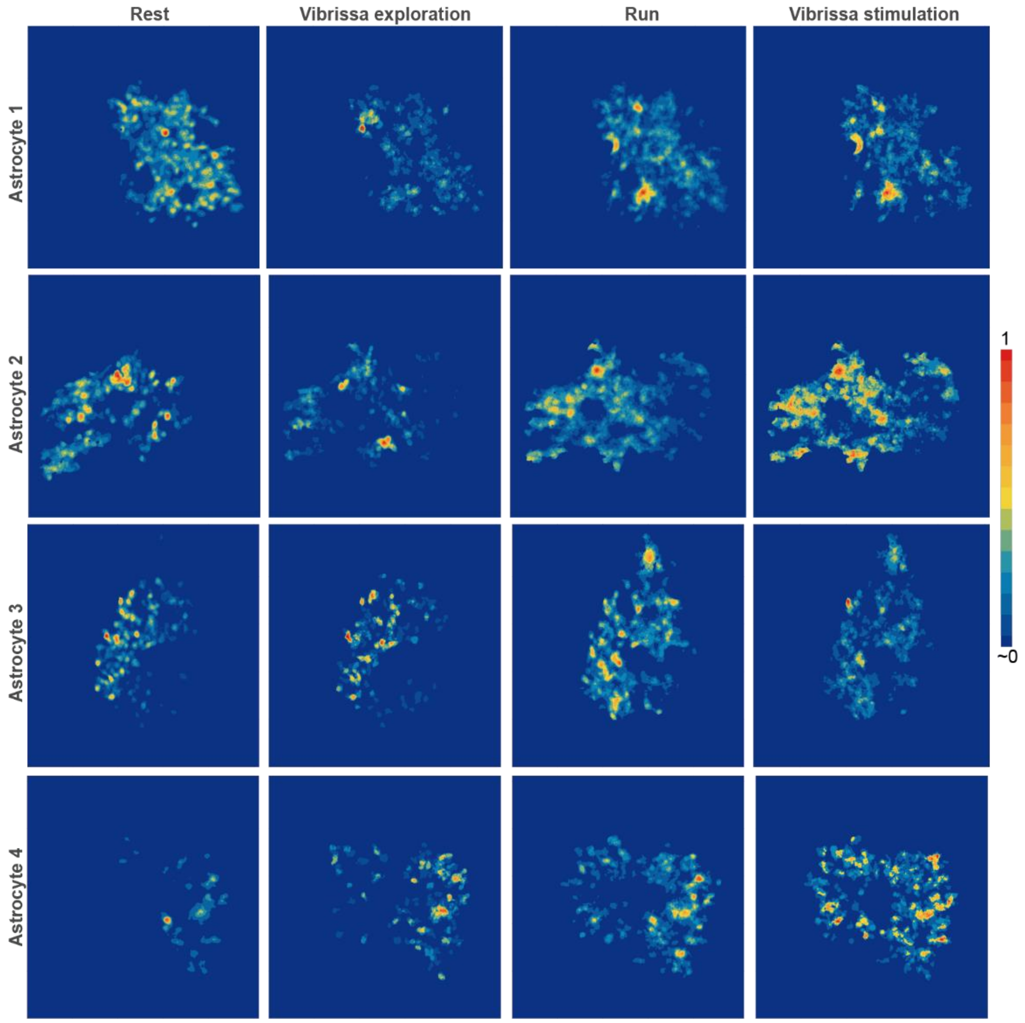

Fig. S10. State-dependent heatmaps of four astrocytes

Normalized activity heatmaps of four astrocytes, generated from Ca^{2+} MD signals evoked during four behavioral states: rest, vibrissa exploration, run, and vibrissa stimulation. Each heatmap is normalized to its own maximum pixel value.

Table S1: Overview of strategies applied to test AAV transfer.

Experiment	AAV constructs injected	Observed outcome	Fig.
Intersectional strategy:<br><br>Anterograde axo-astrocytic AAV transfer	In VPM:<br>AAV1-hSyn-TurboRFP<br>AAV1-CMV-Cre<br><br>In BX:<br>AAV5-GFaABC1D-FLEx-lck-GCaMP6f	Labeling of thalamic neurons and their axonal projections to BX (red).<br><br>Sparse labeling of astrocytes in BX (green) after anterograde axo-astrocytic AAV transfer of AAV1-CMV-Cre to BX neurons transfected by AAV5-GFaABC1D-FLEx-lck-GCaMP6f.<br><br>AAV1-hSyn-TurboRFP is expected to anterogradely transfer to astrocytes and neurons. Due to the hSyn promoter, TurboRFP is expected to be expressed in BX neurons. However, the expression level was not high enough to be detected.	1B<br>2A<br>3B<br>S2A<br>S3A
Intersectional strategy:<br><br>Anterograde axo-neuronal AAV transfer	In VPM:<br>AAV1-hSyn-TurboRFP<br>AAV1-CMV-Cre<br><br>In BX:<br>AAV9-hSyn-FLEx-GCaMP6f	Labeling of thalamic neurons and their axon projections in the BX (red).<br><br>Sparse labeling of neurons in BX (green) after anterograde axo-astrocytic AAV transfer of AAV1-CMV-Cre to BX astrocytes transfected by AAV9-hSyn-FLEx-GCaMP6f.<br><br>AAV1-hSyn-TurboRFP is expected to anterogradely transfer to astrocytes and neurons. Due to the hSyn promoter, TurboRFP is expected to be expressed in BX neurons. However, the expression level was not high enough to be detected.	1C,<br>S2C<br>S3D
Simple strategy:<br><br>Anterograde axo-neuronal and axo-astrocytic AAV transfer	In VPM:<br>AAV1-hSyn-TurboRFP<br>AAV1-CAG-GCaMP6f	Labeling of both neurons and astrocytes in the BX (green) due to the very strong and ubiquitous CAG promoter after anterograde axo-neuronal and axo-astrocytic AAV transfer of AAV1-CAG-GCaMP6f<br><br>AAV1-hSyn-TurboRFP is expected to anterogradely transfer to astrocytes and neurons. Due to the hSyn promoter, TurboRFP is expected to be expressed in BX neurons. However, the expression level was not high enough to be detected.	S1A
Combinatorial strategy:<br><br>Anterograde axo-neuronal and axo-astrocytic AAV transfer	In VPM:<br>AAV1-CMV-Cre<br>AAV1-CAG-Flex-eGFP	Labeling of thalamic neurons and their axon projections in the BX (green). Labeling of thalamic astrocytes (green).<br><br>Labeling of both neurons and astrocytes in BX (green) after anterograde axo-neuronal and axo-astrocytic AAV transfer of AAV1-CMV-Cre and AAV1-CAG-FLEx-GCaMP6f.	S1B
	In VPM:<br>AAV1-CMV-Cre<br>AAV1-CAG-FLEx-tdTomato	Labeling of thalamic neurons and their axon projections in the BX (red). Labeling of thalamic astrocytes (red).<br><br>Labeling of both neurons and astrocytes in BX (red) after anterograde axo-neuronal and axo-astrocytic AAV transfer of AAV1-CMV-Cre and AAV1-CAG-FLEx-tdTomato.	1D-H
Combinatorial strategy:<br><br>Anterograde axo-neuronal and axo-astrocytic AAV transfer	In VPM:<br>AAV1-hSyn-TurboRFP<br>AAV1-CMV-Cre<br>AAV1-CAG-FLEx-GCaMP6f	Labeling of thalamic neurons and their axons projections to BX with GCaMP6f (green) and TurboRFP (red). Labeling of thalamic astrocytes (green).<br><br>Labeling of both neurons and astrocytes with GCaMP6f in BX (green) after anterograde axo-neuronal and axo-astrocytic AAV transfer of AAV1-CMV-Cre and AAV1-CAG-FLEx-GCaMP6f.<br><br>AAV1-hSyn-TurboRFP is expected to anterogradely transfer to astrocytes and neurons. Due to the hSyn promoter, TurboRFP is expected to be expressed in BX neurons. However, the expression level was not high enough to be detected.	S6A,<br>B

Supplementary Movie Captions

Movie S1. Astrocyte Ca^{2+} signaling during different behavioral states.

(Left) The mouse is transitioning between 4 behavioral states (rest, run, vibrissa stimulation, and vibrissa exploration) in real time. The vibrissa stimulus can be identified as the rod intercepting the vibrissae of the mouse.

(Middle) Astrocyte labelled with GCaMP6f tagged to the plasma membrane. This panel shows the raw Ca^{2+} MD activity of the astrocyte synchronized to the mouse behavior on the left.

(Right) Astrocyte Ca^{2+} MD signals shown as false color 2D footprints extracted using AQuA.

Movie S2. Axon-astrocyte interactions

Sample two-photon imaging (291.85s, sped up 6.5x) of axon and astrocyte Ca^{2+} signals in L2/3 of BX. A BX astrocyte is labelled with GCaMP6f (green) and thalamocortical axons (VPM to BX) are labelled with GCaMP6f and TurboRFP (red).

Movie S3. Ca^{2+} activity heatmap generation in an astrocyte.

Progressive summation of Ca^{2+} MD signals extracted from 20 min of activity recorded while the mouse was at rest (left) or running (right). Stable hotspot patterns emerge over time.
